# An intelligent IoT–machine learning framework for wildfire detection and prediction using a hybrid RF–XGB model

**DOI:** 10.1038/s41598-026-52395-w

**Published:** 2026-05-13

**Authors:** Ahmed A. Radhi, Abdullahi A. Ibrahim

**Affiliations:** 1 Electrical and Computer Engineering Department, Graduate School of Science, Altinbas University, Istanbul, 34217 Turkey; 2 Department of Artificial Intelligence and Robotics, College of Engineering, Al-Nahrain University, Baghdad, 64040 Iraq

**Keywords:** IoT, WSN, Wildfire, Forest fire prediction, Forest fire detection, AI, RF-XGB, Engineering, Mathematics and computing

## Abstract

Forest fires in Turkey have received comparatively limited scholarly attention despite the country’s high seasonal susceptibility, particularly during summer due to adverse climatic conditions. For real-time detection and risk assessment, this research suggests an integrated intelligent wildfire monitoring and prediction framework that integrates a unique weighted-voting RF-XGB hybrid model with Internet of Things (IoT)-based wireless sensor networks (WSNs). The adaptive weighting approach, which goes beyond traditional majority-voting ensembles, combines Random Forest and Extreme Gradient Boosting to take use of complementary variance-reduction and boosting processes. This is the methodological innovation. A multi-season Turkish forest fire dataset that included environmental sensor data, including temperature, relative humidity, and carbon monoxide concentration, was used to train the model. Distribution-preserving sampling and stratified k-fold cross-validation were used to alleviate class imbalance. With an accuracy of 0.9631, F1-score of 0.9627, and ROC–AUC of 0.994, the suggested hybrid model outperforms the others when compared to RF, XGBoost, KNN, Decision Tree, MLR, SVM, and ANN. Larger improvements were shown over KNN (10.4%) and Decision Tree (18.3%), while Relative Improvement (RI), as determined by the AUC measure, reveals a 4.6% increase over XGBoost and 5.7% over Random Forest—the strongest baselines. When compared to MLR, SVM, and ANN, improvements of over 50% were seen, demonstrating the hybrid model’s greater robustness and discriminating capabilities. At the system level, a lightweight Multiple Logistic Regression (MLR) model was deployed on Arduino Nano-based sensor nodes to enable edge-level probability estimation and reduce communication overhead. Nodes operate using hourly duty cycling and transmit only when fire probability exceeds a predefined threshold, achieving an analytically estimated lifetime of up to 11 months. The framework was implemented in Zeytinpark using 80 sensor nodes deployed via hybrid grid and K-means clustering, achieving 95.58% coverage. Real-time detections are verified at the sink node using the RF-XGB model before triggering multi-level alerts, including local alarms, cloud updates, Telegram notifications, and mobile-based fire localization. The results demonstrate that the proposed contribution lies in the adaptive hybrid ensemble design, hierarchical edge–cloud intelligence distribution, and validated real-world deployment. The framework provides a robust, energy-efficient, and scalable solution for rapid wildfire detection and forecasting.

## Introduction

Forests are vital ecosystems that maintain biodiversity, control the temperature, and lessen environmental hazards like soil erosion and floods. Additionally, they are crucial for preserving environmental stability and sequestering carbon^1^. Despite their significance, forest fires continue to rank among the most damaging natural disasters because of their quick spread and serious ecological and financial repercussions, which include damage to infrastructure, biodiversity loss, and air pollution^2^. Natural events like lightning and dryness, as well as human-caused events like uncontrolled fires, industrial processes, and electrical infrastructure, may start wildfires^3,4^.

Numerous regional and global organizations have created monitoring systems based on satellite views and data-driven analysis to aid in wildfire control and detection in response to the rise in wildfire incidents^5–12^. These programs emphasize how crucial it is to have automated and intelligent monitoring systems that can facilitate early identification and prompt action at the local level.

**Fig. 1:**
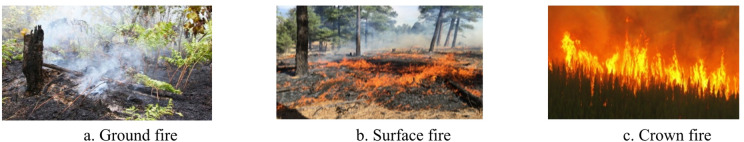
Forest fire classification.

In recent years, wildfires have become more intense worldwide, resulting in substantial losses to the environment and human life^13–15^. Crown, surface, and ground fires are among the wildfire types shown in Fig. 1, which emphasizes the complexity of fire behavior and the need for multi-sensor monitoring techniques. Descriptive data of wildfire losses recorded in prior years are also included in Table 1. When information was unavailable or not recorded in the cited sources, some fields are indicated as “NA.”

An increasingly important part of managing and preventing wildfires is mapping high-risk regions^16,17^. Accurate prediction is still difficult in wildfire-prone areas like southern Turkey, and there is currently no research combining real-time sensing with sophisticated machine learning techniques. Using human and environmental aspects for prediction, previous research has investigated a variety of machine learning techniques, such as Decision Trees, Random Forest, Logistic Regression, Artificial Neural Networks, Support Vector Machines, K-Nearest Neighbors, and Extreme Gradient Boosting.

Data-driven wildfire research and risk mapping are supported by contemporary systems, including the European Forest Fire Information System, Google Earth Engine, and the Environment for Visualizing Images (ENVI)^18^. Instead of integrating energy-efficient edge intelligence into real-time wireless sensor networks, many existing methods primarily depend on centralized processing or distant sensing analysis. This research suggests an integrated intelligent wildfire monitoring system that integrates hybrid machine learning approaches with wireless sensor networks based on the Internet of Things (IoT) in order to overcome these constraints. Lightweight local inference is carried out by sensor nodes, and precise fire risk prediction at the sink/cloud level is achieved via a weighted hybrid model that combines Random Forest and Extreme Gradient Boosting. In real-world deployment scenarios, the suggested system seeks to increase monitoring efficiency, decrease false alarms, and improve early wildfire detection by combining real-time sensing, edge-level intelligence, and hybrid AI modeling into a single framework.

**Table 1:** Forest fires losses for the period from 2021 to 2025.

Geographic location	Deaths	Injuries	Area burned (ha)	Damaged trees	Affected wildlife	Year	Country
Manavgat, Gündoğmuş, Marmaris, Bodrum, Köyceğiz, Milas	8–9	> 800	140,000–202,361	Worst season; destruction of pine, olive groves, biodiversity loss	Thousands of animals perished	2021	Türkiye
Fethiye, Seydikemer, Antalya, Marmaris	N/A	N/A	12,384	Reduced fire severity compared to 2021	Not reported	2022	Türkiye
Aragón region	N/A	N/A	306,555	Extensive forest canopy loss	Bird and small animal fatalities	2022	Spain
Antalya Province, Mersin, Muğla, Aydın	N/A	N/A	~ 15,000	Fire incidence continued decline	Not reported	2023	Türkiye
Rhodes Island	28	75	175,000	Mediterranean forest destruction	Thousands of wild animals perished	2023	Greece
Antalya (Manavgat, Gündoğmuş), Muğla, Aydın	12+	78+	~ 27,000	extensive forest and agricultural damage	Hundreds of livestock killed	2024	Türkiye
Jasper National Park, Alberta	1	N/A	39,000	Mixed pine/spruce forests destroyed	Destroyed habitats: bats, bears, deer	2024	Canada
İzmir & Antalya regions	2	N/A	35,000	Olive and pine forests burned	Significant bird and small animal deaths	2025	Türkiye
Palisades, California	12	4	9,500	Thousands of ancient trees lost	Impact on migratory birds and mammals	2025	United States
Los Angeles	18	8	5,680	Entire coniferous forests burned	Wildlife loss: lizards, small mammals	2025	United States

## Related works

Machine learning methods have been crucial in the last several years for stopping and treating forest fires. Dwiasnati and Devianto, for instance, suggested using different machine learning techniques to figure out where forest fires are happening^19^. Pang et al. also recommended employing a set of machine learning algorithms to forecast forest fire occurrences in China^20^. Dampage et al. suggested using wireless sensor networks and machine learning algorithms to interpret data to find forest fires^21^. Shao et al. suggested utilizing a number of machine learning algorithms to map China’s forest fire hazards^22^. Singh et al.^23^ proposed a parallel SVM model for predicting forest fires, utilizing data gathered from India and Portugal. Hybrid feature selection techniques have been widely used to improve classification performance by identifying the most informative variables while reducing redundancy. Ram Ji et al.^24^. proposed a hybrid feature selection framework that enhances detection accuracy and computational efficiency. Although the study targets intrusion detection systems, the underlying feature optimization concept is relevant to machine learning–based wildfire risk prediction models. Fire risk modeling using advanced machine learning techniques has gained increasing attention in recent years. Mert Nakıp et al.^25^ proposed a Support Vector Regression (SVR) framework with augmented regularization to improve fire detection and risk assessment performance. The study demonstrated that enhanced regularization strategies can improve generalization capability and predictive stability in fire-related modeling tasks. Deep learning approaches have also been widely applied to wildfire detection tasks. Seon Ho Oh et al.^26^ proposed a convolutional neural network (CNN)-based framework for early wildfire detection using visual data. The study demonstrated the effectiveness of deep feature extraction in identifying fire-related patterns at early stages. Such vision-based deep learning methods complement data-driven environmental and machine learning models used in wildfire risk prediction. To evaluate the performance of the proposed hybrid model, several well-established machine learning algorithms were implemented for comparison, including Decision Tree (DT)^27^, Random Forest (RF)^28^, Logistic Regression (LR)^29^, Artificial Neural Network (ANN)^30^, Support Vector Machine (SVM)^31^, K-Nearest Neighbors (KNN)^32^, and extreme gradient boosting (XGBoost)^33^ in addition to new hybrid AI-model that proposed in this study for forest fire detection. All comparative models were trained using the same input features and evaluation metrics to ensure a fair comparison.

## Materials and methods

IoT-based fire detection and data-driven modeling were combined to provide a thorough methodological framework. In order to assess their prediction capabilities and determine the best method for wildfire risk modeling, a number of machine learning algorithms were put into practice and contrasted. A new hybrid model was created to increase prediction accuracy and spatial dependability based on the comparative findings. In order to promote early warning and effective forest fire management, the suggested hybrid model was then connected with an Internet of Things (IoT) platform to provide real-time wildfire detection and monitoring.

### Study area

**Fig. 2:**
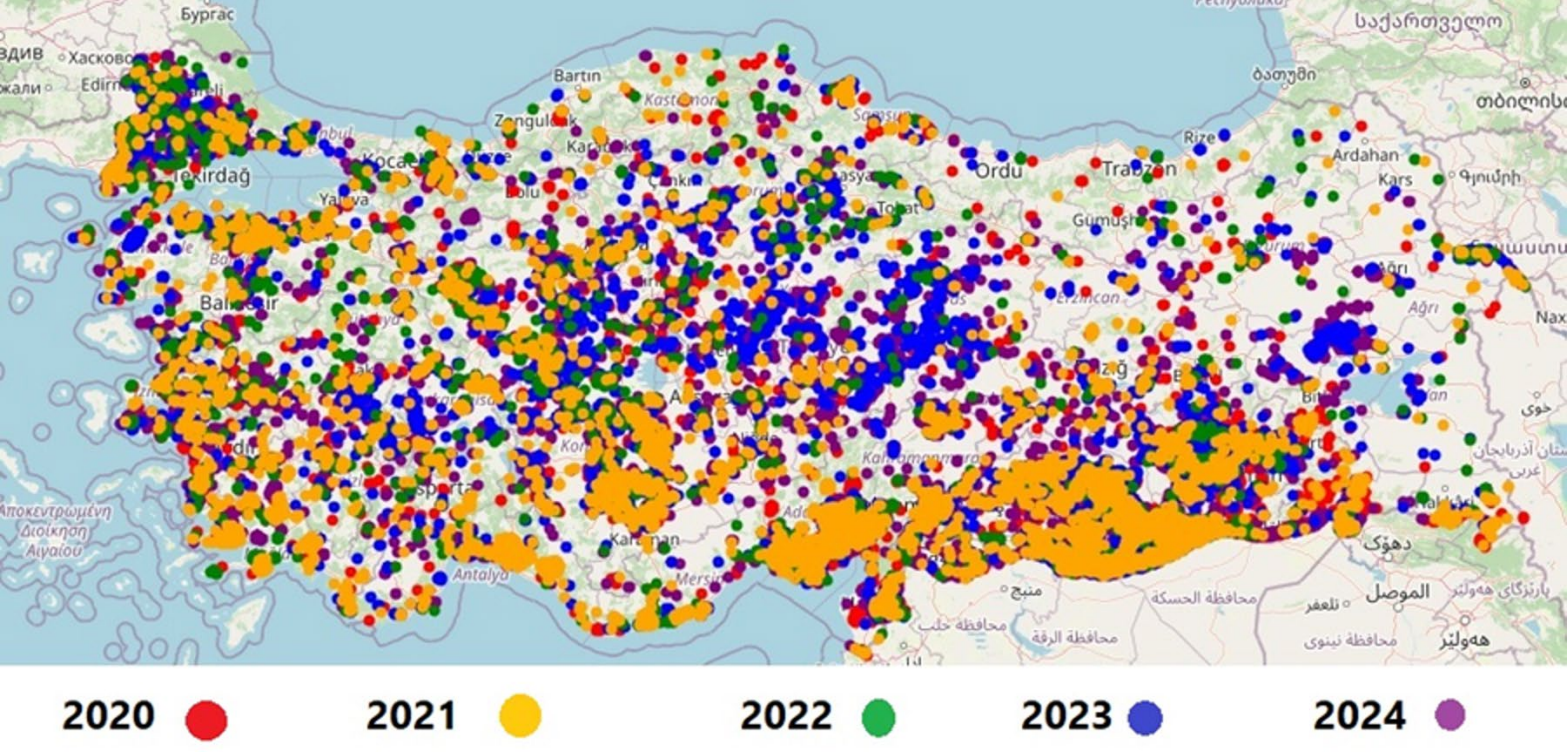
Recorded land fires of Turkey between 2020 and 2024.

Turkey is distinguished by its geographical location, lying between the continents of Asia and Europe, and its proximity to a group of seas, which gives it a unique climate, characterized by dry summers and mild winters in the south and on the western coast, a continental climate in the interior, and an oceanic climate in the north along the Black Sea coast. This diversity has made it rich in forests, especially in the southwest (the regions of Antalya, Mersin, Muğla, Izmir…). In recent years, especially between 2020 and 2023, Turkey has witnessed waves of large forest fires. The most prominent of these were the summer 2021 fires in the regions of Antalya, Muğla, Mersin, and others, which were considered among the worst environmental disasters in the country’s history. This was due to climate change (high temperatures and drought), strong winds, and ill-considered human activities (such as agricultural burning). Figure 2, shows the fire areas in Türkiye for the period from 2020 to 2024, which shows that most of the fires are concentrated in the forests of southern Türkiye, according to the National Aeronautics and Space Administration (NASA).

**Fig. 3:**
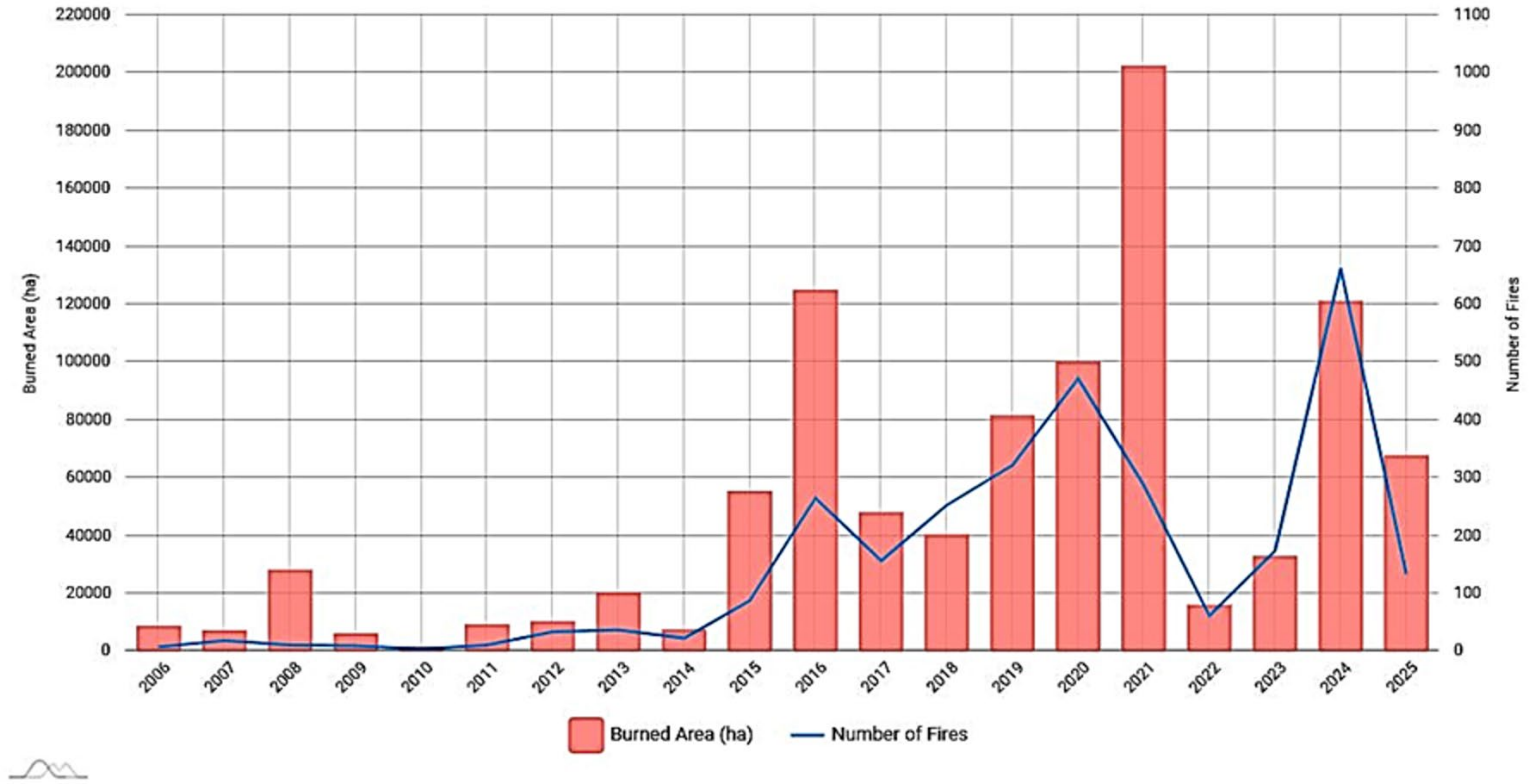
Recorded number of fires burned area in Turkey between 2006 and 2024.

Figure 3, shows the number of fires and the affected areas in Türkiye for the period from 2006 to 2024, which shows most of the fires are concentrated according to the European Forest Fire Information System (EFFIS).

**Fig. 4:**
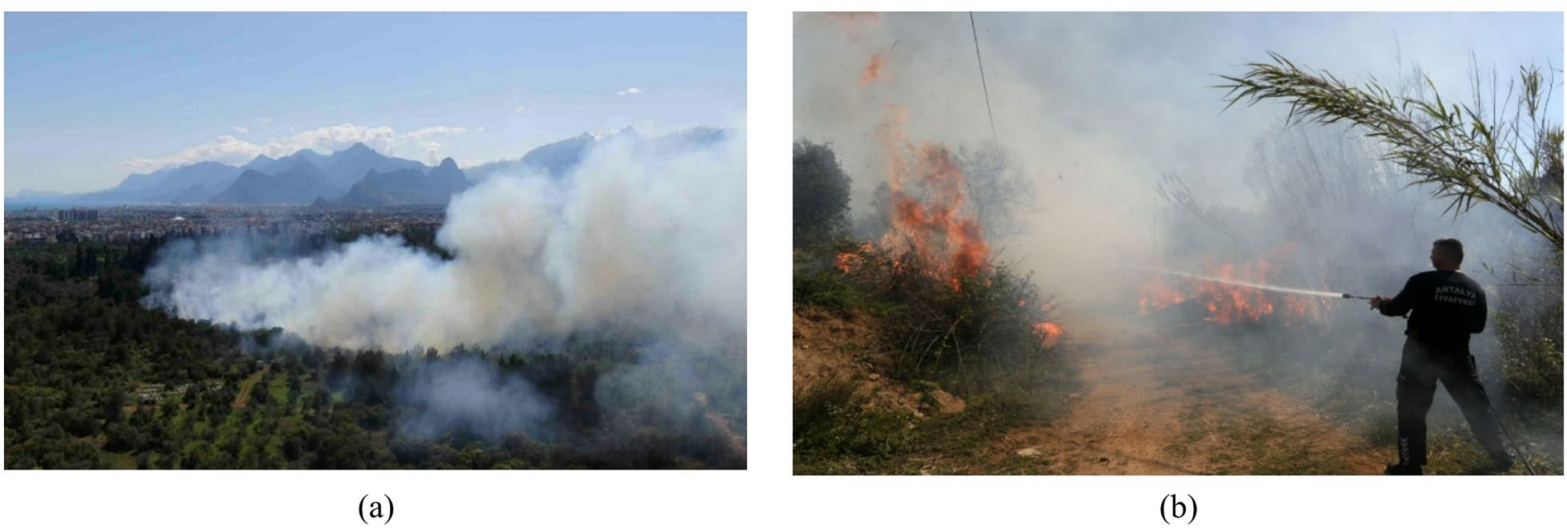
Zeytinpark fire treatment^34^.

Zeytinpark, located in the Kepez-Ahatlı district of Antalya, is a large green space within the city, considered the “lungs” of Antalya. It covers a total area of approximately 2,630,000 square meters. The park contains around 25,000–26,000 trees, mostly olive trees, along with pine and other native species. These characteristics make Zeytinpark an important environmental, social, and economic part of the city, serving as an educational, agricultural, and tourist site for the region. The site has been affected by fire in previous years, most recently on April 13, 2025. A fire broke out in the central part of Zeytinpark, requiring the intervention of firefighters and other relevant authorities. The fire was brought under control after intensive efforts. It should also be noted that the area contains tens of thousands of olive trees, making it highly susceptible to fire hazards during the summer months and exposing it to both production and environmental losses. The repeated occurrence of fires underscores the need for an early warning infrastructure. Local media have documented live images of these fires, including the smoke rising and the on-site firefighting operations, providing real-world references for using the site as an applied model for an IoT-based fire detection system (Fig. 4).

### Dataset

The dataset was utilized to develop and compare wildfire prediction models. Publicly available datasets, including **NASA FIRMS (MODIS and VIIRS Active Fire)** [35] provide near real-time global fire detection data derived from the Moderate Resolution Imaging Spectroradiometer (MODIS) and Visible Infrared Imaging Radiometer Suite (VIIRS) instruments. The dataset includes active fire points, brightness temperature, confidence level, and detection time, covering the period from 2000 to 2024. Fire presence points extracted from NASA FIRMS were complemented with dynamically generated absence (non-fire) points to construct a balanced binary classification dataset. The dataset contains thousands of labeled samples including spatial–temporal attributes (latitude, longitude, acquisition date) and environmental features such as temperature, relative humidity, and carbon monoxide concentration [36]. The FIRMS fire records are publicly available datasets, while sensor measurements collected from the deployed WSN in Zeytinpark were generated during this study for real-time validation purposes.

### Dataset preprocessing

Data preprocessing is an essential step in machine learning workflows, as it improves data quality and model performance. In this study, several preprocessing techniques were applied before training. First, categorical data encoding was performed for the target variable, which consists of two classes: fire and non-fire. Label encoding was used to convert class labels into numerical values suitable for model processing. In addition, one-hot encoding was applied to categorical features such as the daynight variable, where daytime fires were represented as 1 and nighttime fires as 0, as defined in Eq. (1).

1 $$\:Daynight=\left\{\begin{array}{c}1\to\:\:for\:daytime\:fire\:\\\:0\:\to\:\:for\:nighttime\:fire\end{array}\right.$$

Data cleaning was then performed to handle potential errors, duplicates, and missing values in the raw dataset [37]. Missing values were replaced using the mean value method, which was selected due to its simplicity and effectiveness for continuous environmental variables, as shown in Eq. (2).

2 $$\:Mean=\frac{\sum\:_{i=1}^{n\:}{x}_{i\:}}{n}$$

Furthermore, feature normalization was applied to scale numerical features into a unified range between 0 and 1. This step improves model stability and convergence by preventing features with larger numerical ranges from dominating the learning process. The normalization process is defined in Eq. (3).

3 $$\:{{X}^{{\prime\:}}}_{i}=\frac{{X}_{i}-{X}_{min}}{{X}_{max}-{X}_{min}}$$

Finally, feature engineering was conducted using the Turkey fire dataset (2000–2021), which includes fire locations represented by latitude and longitude. Additional fire data from 2021 to 2024 were obtained from the NASA portal. A balanced dataset with fire and non-fire classes was produced by creating non-fire points based on the temporal absence of fire records at the same locations. To improve prediction accuracy, other environmental factors were included, such as temperature, relative humidity, and carbon monoxide content. To lessen class imbalance and guarantee a balanced dataset for training and assessment, non-fire (absence) points were created utilizing temporal and geographical criteria. Under significant class imbalance, this method promotes dependable learning and lessens prejudice toward the dominant class.

The manuscript has been revised to clarify the data splitting strategy. An 80%–20% stratified split was used to create independent training and testing sets. In addition, k-fold cross-validation was applied within the training set to ensure robust model validation and improve generalization. This clarification has been added to the revised manuscript. To further handle class imbalance, stratified data splitting was applied to preserve class distribution in training and testing sets. Model evaluation emphasizes imbalance-aware metrics such as ROC–AUC and F1-score in addition to accuracy.

### Machine learning techniques

In this study, baseline performance results for all compared models (RF, XGBoost, ANN, SVM, and MLR) are explicitly reported and compared using the same evaluation criteria to ensure transparent benchmarking of the proposed RF–XGB hybrid model. Although previous studies have successfully applied individual machine learning models for forest fire prediction, most of them focus either on centralized processing or standalone algorithms without considering resource-constrained IoT environments. In contrast, this study introduces a hybrid edge–cloud intelligence framework that combines lightweight MLR inference at sensor nodes with a hybrid RF–XGB model at the sink/cloud level. This architectural approach tackles computational constraints, energy economy, and real-time operation in IoT-based wireless sensor networks in addition to increasing prediction accuracy using hybrid learning. To provide fair and transparent benchmarking, all baseline models are assessed using the same dataset, feature set, and assessment criteria, in contrast to previous efforts.

### IoT

**Fig. 5:**
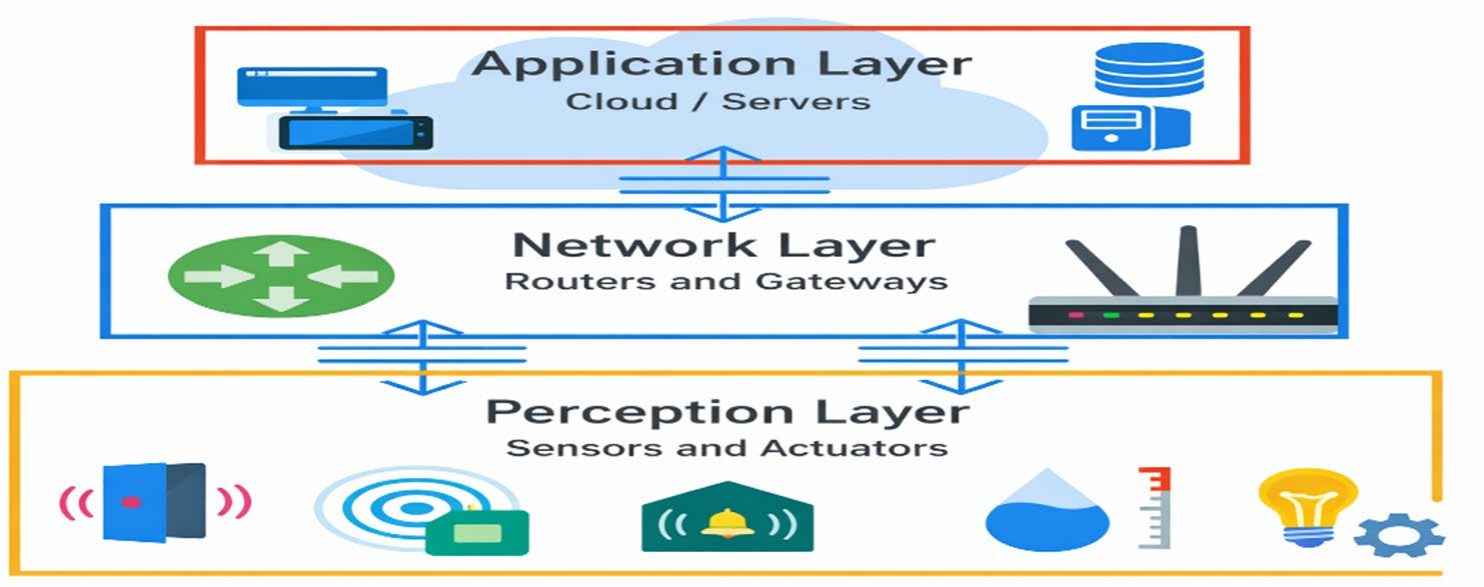
IoT architecture.

The Internet of Things (IoT) enables connected devices and sensors to exchange data through the internet and supports remote monitoring via cloud-based platforms [38]. Due to advances in web applications and embedded electronics, IoT has been widely adopted in monitoring and automation systems, including environmental sensing applications [39]. The IoT architecture typically consists of three layers: perception, network, and application layers, where sensors collect environmental data, communication networks transfer data, and application services perform analysis and decision-making tasks [40]. In IoT-based wireless sensor networks (WSNs), the perception layer includes sensor nodes that continuously monitor environmental conditions and transmit data to a sink node or gateway for further processing (Fig. 5).

In this study, the IoT architecture is specifically designed for wildfire monitoring using resource-constrained sensor nodes connected to a sink/cloud infrastructure. Sensor nodes perform local data acquisition and lightweight inference, while advanced processing is executed at the sink/cloud level. To optimize edge–cloud intelligence distribution, Multiple Logistic Regression (MLR) is deployed at sensor nodes due to its minimal computational and memory requirements. MLR relies on simple linear operations with sigmoid activation, enabling real-time inference on Arduino Nano microcontrollers (32 KB flash, 2 KB SRAM, 16 MHz CPU). This lightweight implementation reduces energy consumption and supports duty-cycling, extending node lifetime up to 11 months. More complex analysis is performed at the sink/cloud level using the hybrid RF–XGB model, which provides robust verification and improved prediction accuracy. The cloud database is updated based on model outputs, including fire warnings and fire location information.

## Proposed model (RF-XGB)

**Fig. 6:**
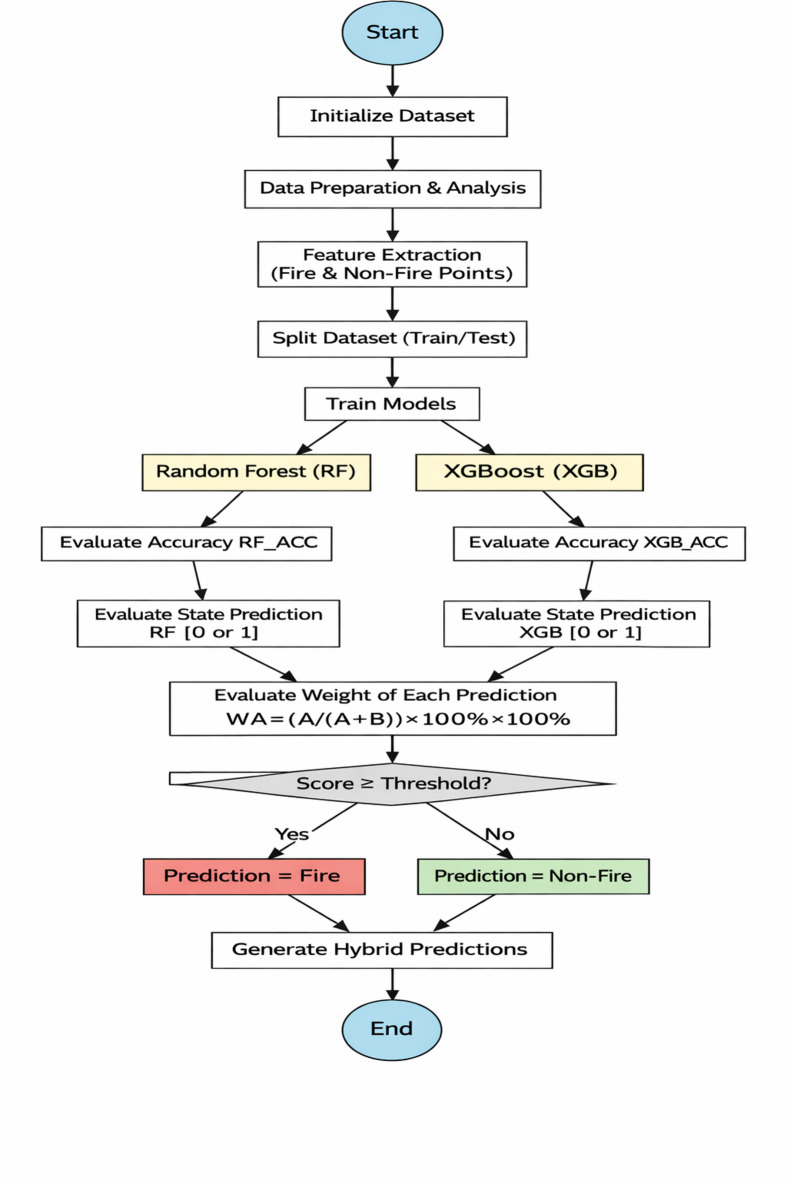
Algorithm flowchart of the proposed model (RF-XGB).

The dataset used depends on several temporal, spatial, and other environmental factors. To improve the training and prediction process, an algorithm that handles geographic field data well, handles unbalanced data, and is strong at generalizing and distinguishing between fires and non-fires is needed. XGBoost (Extreme Gradient Boosting) is very accurate even with complex patterns, handles feature interactions and missing values well, and works great with tabular wildfire data. Random Forest is very stable and noise-resistant. It performs well even with small parameter tuning, easy to interpret and visualize. The hybrid model is a combination of both algorithms that works based on weighted voting prediction. The combination yields high accuracy overall, with a balanced output that includes stability, robust predictions, easy interpretation, and full coverage of the dataset, as illustrated in Fig. 6.

4 $$\:{W}_{RF}=\frac{{RF}_{Acc}}{{RF}_{Acc}+{XGB}_{Acc}}\times\:100\%$$

5 $$\:{W}_{XGB}=\frac{{XGB}_{Acc}}{{XGB}_{Acc}+{RF}_{Acc}}\times\:100\%$$

6 $$\:Fire\:Score=\left({W}_{XGB}\times\:{XGB}_{pred}\right)+\left({W}_{RF}\times\:{RF}_{pred}\right)$$

7 $$\:Propability\:of\:fire=\left\{\begin{array}{c}Fire\:Score\ge\:\:\omega\:\:\to\:1\:\left(high\:risk\:fire\right)\\\:Fire\:Score<\:\omega\:\:\to\:0\:\:\:\:\:\:\left(non-\:fire\right)\end{array}\right.$$

where W_RF_ is the weight of the RF algorithm, W_XGB_ is the weight of the XGB algorithm, RF_ACC_ is the accuracy of the RF, and XGB_ACC_ is the accuracy of the XGB algorithm. If the Fire Score is larger than or equal to ω, then we have a forest area with high-risk fire, where the threshold value is (0 ≤ ω ≤ 1).

Preliminary trials and widely accepted values from earlier wildfire prediction research were used to manually configure the Random Forest (RF) and XGBoost (XGB) models’ hyperparameters. In order to balance computing performance and model complexity, a number of parameter combinations were first evaluated. While the XGBoost model was set up with learning_rate = 0.1, n_estimators = 100, and max_depth = 15, the final RF model was trained with n_estimators = 100. To guarantee uniform assessment across several geographic folds, these parameters were maintained constant throughout the spatial 10-fold cross-validation procedure.

The RF–XGB model was calibrated using Turkey-specific environmental and climatic conditions. While the trained model parameters are region-specific, the proposed hybrid modeling framework and IoT-WSN architecture are transferable and can be adapted to other regions by retraining using local datasets.

## Evaluation metrics

- **models performance**: Some criteria are used to evaluate the models performance in detecting and prediction of forest fires, including accuracy (ACC), which represents the overall performance of the proposed model, precision (Pre), which represents the ratio of positive predictions to the total predicted positive class, recall (RCC), also known as sensitivity or true positive rate measures the proportion of actual fire events that are correctly identified by the model, and the (F1).

8 $$\:\mathrm{A}\mathrm{c}\mathrm{c}\mathrm{u}\mathrm{r}\mathrm{a}\mathrm{c}\mathrm{y}\:=\:\left(\right(\mathrm{T}\mathrm{P}\hspace{0.17em}+\hspace{0.17em}\mathrm{T}\mathrm{N}\left)\right)/\left(\right(\mathrm{T}\mathrm{P}\hspace{0.17em}+\hspace{0.17em}\mathrm{F}\mathrm{P}+\mathrm{T}\mathrm{N}\hspace{0.17em}+\hspace{0.17em}\mathrm{F}\mathrm{N}\left)\right)$$

9 $$\:\mathrm{P}\mathrm{r}\mathrm{e}\mathrm{c}\mathrm{i}\mathrm{s}\mathrm{i}\mathrm{o}\mathrm{n}\:=\:\mathrm{T}\mathrm{P}/\left(\right(\mathrm{T}\mathrm{P}+\mathrm{F}\mathrm{P}\left)\right)$$

10 $$\:\mathrm{R}\mathrm{e}\mathrm{c}\mathrm{a}\mathrm{l}\mathrm{l}\:=\:\mathrm{T}\mathrm{P}/\left(\right(\mathrm{T}\mathrm{P}+\mathrm{F}\mathrm{N}\left)\right)$$

11 $$\:\mathrm{F}1\mathrm{S}\mathrm{c}\mathrm{o}\mathrm{r}\mathrm{e}\:=\:\left(\right(2\times\:\mathrm{P}\mathrm{r}\mathrm{e}\mathrm{c}\mathrm{i}\mathrm{s}\mathrm{i}\mathrm{o}\mathrm{n}\times\:\mathrm{R}\mathrm{e}\mathrm{c}\mathrm{a}\mathrm{l}\mathrm{l}\left)\right)/\left(\right(\mathrm{P}\mathrm{r}\mathrm{e}\mathrm{c}\mathrm{i}\mathrm{s}\mathrm{i}\mathrm{o}\mathrm{n}+\mathrm{R}\mathrm{e}\mathrm{c}\mathrm{a}\mathrm{l}\mathrm{l}\left)\right)$$

- **Quantifying relative improvement**:

The relative improvement (RI) of the proposed hybrid model was calculated to quantify the performance gain over a baseline model. It is defined as:

12 $$\:RI\left(\%\right)=\frac{{AUC}_{Hybrid}-{AUC}_{Baseline}}{{AUC}_{Baseline}}\times\:100$$

where $\:AU{C}_{Hybrid}$ denotes the Area Under the Curve of the proposed hybrid model, and $\:AU{C}_{Baseline}$ denotes the AUC of the compared individual model. This metric expresses the performance gain as a percentage, providing a standardized way to evaluate improvement across different baseline models.

- **Confidence Interval (CI)**.

The suggested wildfire detection model’s robustness and statistical dependability are assessed using the confidence interval (CI). The Student’s t-distribution across the cross-validation folds was used in this investigation to get the 95% confidence interval. The definition of the CI is:

13 $$\:CI=\bar{X}\pm\:{t}_{\frac{\alpha\:}{2},n-1}\frac{s}{\sqrt{n}}$$

where $\bar{X}$ is the mean performance measure, s is the standard deviation, n is the number of folds, and $\:{t}_{\frac{\alpha\:}{2}}$, $\:{t}_{\frac{\alpha\:}{2},n-1}$ is the 95% confidence level crucial t-value. Stable and trustworthy wildfire forecast ability is shown by narrow confidence ranges.

- **Paired t-test**: It determines if there is a significant variation from zero in the mean difference between two related samples. The computation of the t-statistic is.

14 $$\:t=\frac{\bar{d}}{{s}_{d}/\sqrt{n}}$$

where $\bar{d}$ is the mean difference, $\:{s}_{d}$ is the number of pairings, and n is the standard deviation of differences. The likelihood that the observed difference happened by accident is indicated by the p-value. The suggested model outperforms the baseline when the p-value is less than 0.05, indicating that the improvement is statistically significant.

- **Power consumption**: The NRF24L01 operates using a compact packet (frame) structure optimized for low-power wireless sensor networks. The module supports a maximum payload size of 32 bytes, in addition to the physical layer overhead, which includes the preamble (1 byte), address field (3–5 bytes), packet control field (2 bytes), and CRC (1–2 bytes). This small frame design reduces radio-on time and helps minimize energy consumption during transmission.

15 $$\:P=\left\{\begin{array}{c}{P}_{sensors}\:\to\:nonfire\\\:{P}_{sensors}+{P}_{TX}\to\:predict\:fire\:of\:Sensor\:node\\\:{P}_{sensors}+{P}_{TX}+{P}_{RX}\to\:predict\:fire\:of\:Sink\:node\end{array}\right.$$

where P is the power consumption of the sensor node, P_sensor_ is the power consumption of the sensors, P_TX_ is the power of transmission packets, and P_RX_ is the power of received packets.

- **Coverage area**: Sensor coverage of forest areas is crucial for building early warning systems for forest fires using the Internet of Things (IoT). The system’s effectiveness depends directly on the sensor network’s ability to monitor thermal and gaseous changes throughout the forest, eliminating blind spots. Complete coverage minimizes response time for detecting initial fire indicators, such as rising temperatures or increased carbon monoxide concentrations, enabling intervention before the fire spreads widely. To achieve effective coverage, recent studies utilize various geometric and mathematical methods for sensor point deployment, including:
- Grid deployment to ensure uniform coverage.
- K-means-based deployment for grouping points and selecting cluster heads to minimize distances between nodes.

The process of calculating optimal coverage relies on a set of mathematical laws, the most important of which are:

16 $$\:A\_node=\pi\:{R}^{2}$$

17 $$\:N=\frac{A\_forest}{A\_node}$$

where (A_node) is the area covered by the sensor node with radius (R), (N) is the number of sensor nodes for covering the area of the forest (A_forest).

## Model and system design

**Fig. 7:**
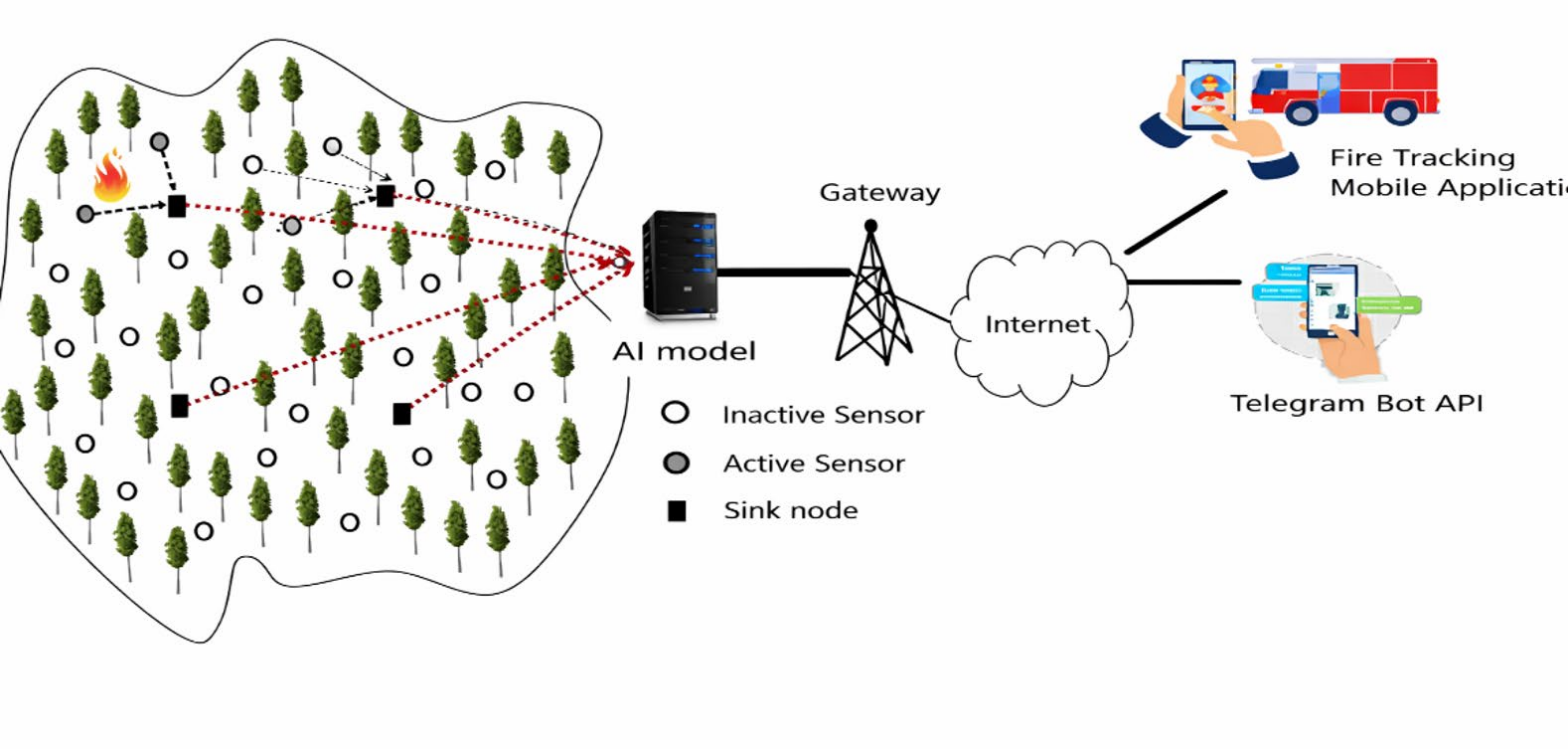
IoT–AI for early wildfire detection and notification.

Both an IoT-based monitoring system and a predictive hybrid model were developed and integrated into a single framework to enable precise and timely wildfire identification. To improve prediction accuracy and stability, the hybrid model combines the advantages of Extreme Gradient Boosting (XGB) and Random Forest (RF). Simultaneously, an Internet of Things (IoT) network was established to collect data from outdoor sensors in real-time regarding temperature, humidity, and carbon monoxide levels. When a sensor detects a possible fire, it sends the information to an AI model to determine whether it is a fire or not. If the detection indicates a fire, the model sends a warning message to all sensors to trigger the alarm. It also sends a warning to firefighters via a phone app, updating the cloud database containing the fire’s location on interactive maps. A warning message will also be sent via the Telegram BOT API to warn people of the fire’s location (Fig. 7).

### Hardware requirements

The proposed system requires a set of hardware components that work together to ensure measurement accuracy and continuous data transmission across different operating environments. The system utilizes an ATmega328P CPU due to its low power consumption and ability to handle sensor data and transmit it to a server or cloud platform by using RF transceiver module (nRF24L01). This includes a set of essential environmental sensors such as a temperature sensor, a humidity sensor (DHT11), and a carbon monoxide (CO) sensor (MQ-7 ), which are crucial for detecting pre-combustion conditions. The system also requires a wireless communication module to ensure data transmission to the network with adequate coverage over large areas. Additionally, the project requires a stable power supply such as a rechargeable lithium battery.

### Software requirements

**Fig. 8:**
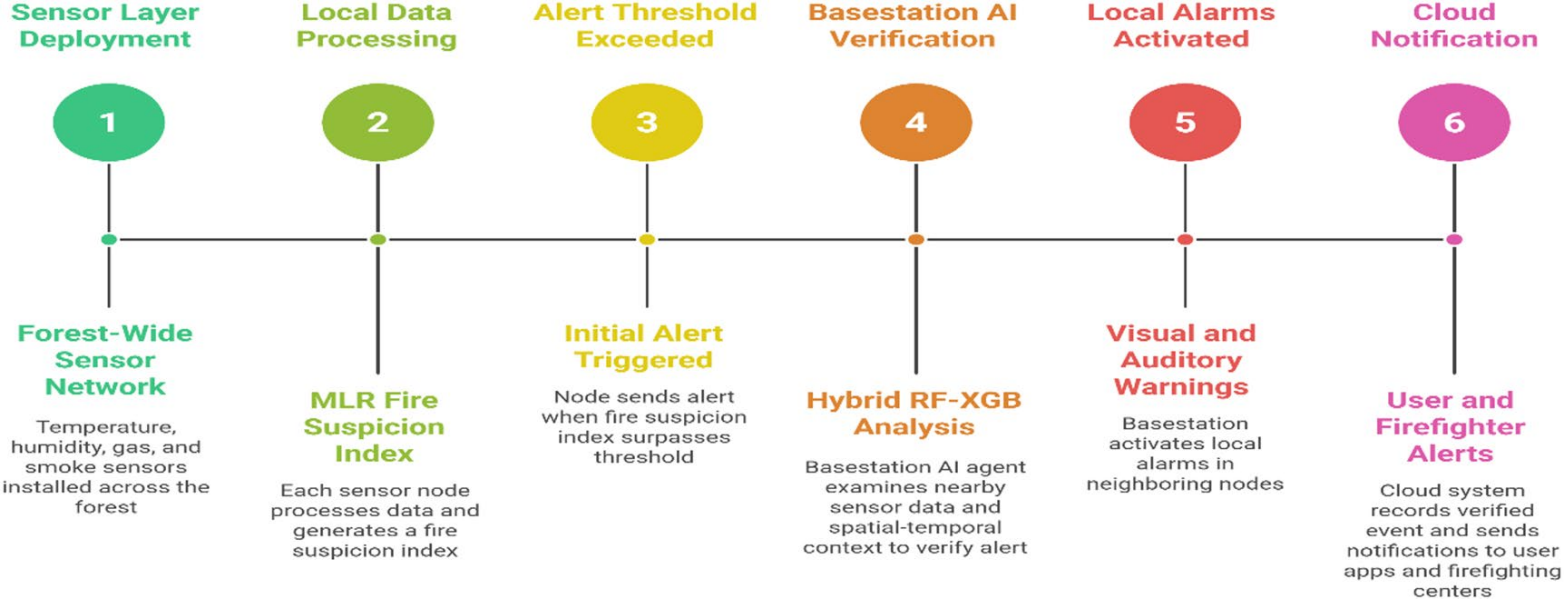
Three-layer forest fire detection system.

The proposed early wildfire detection system uses a wireless sensor network (WSN) with three layers. The sensor layer monitors temperature, humidity, CO, and smoke, using a Multiple Logistic Regression (MLR) model to generate a “fire suspicion” index, sending alerts only when a threshold is exceeded to reduce energy use and network traffic. The gateway layer employs a hybrid RF-XGB AI model to verify alerts and reduce false alarms, activating local alarms when necessary. The cloud layer records confirmed events and sends real-time notifications to users and firefighting teams via a mobile app and Telegram Bot, displaying fire locations on interactive maps (Fig. 8).

### System architecture of the IoT-based forest fire detection network

**Fig. 9:**
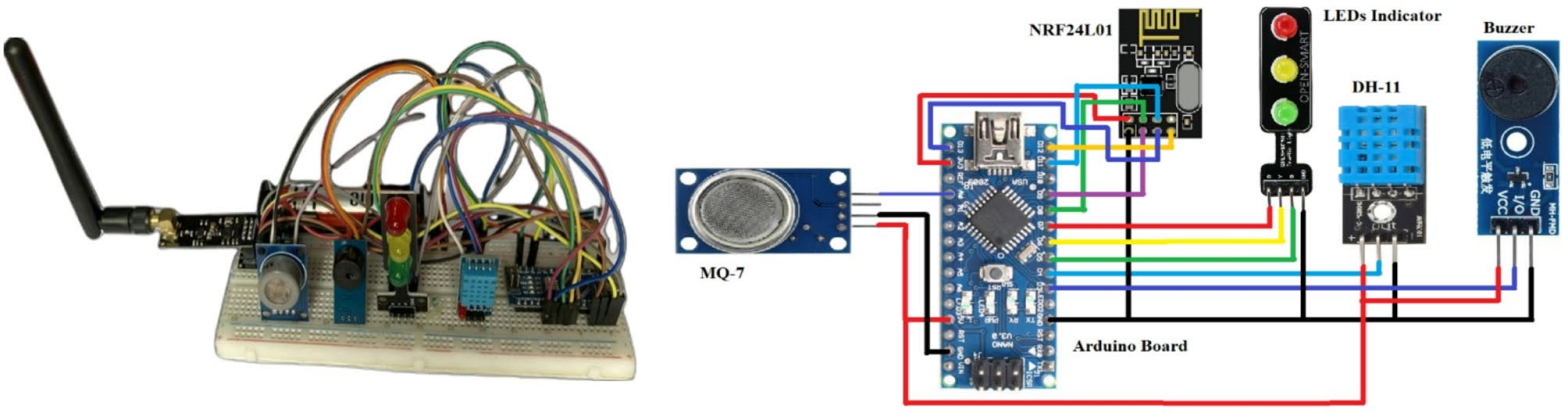
Prototype circuit diagram of the sensor-node.

**Fig. 10:**
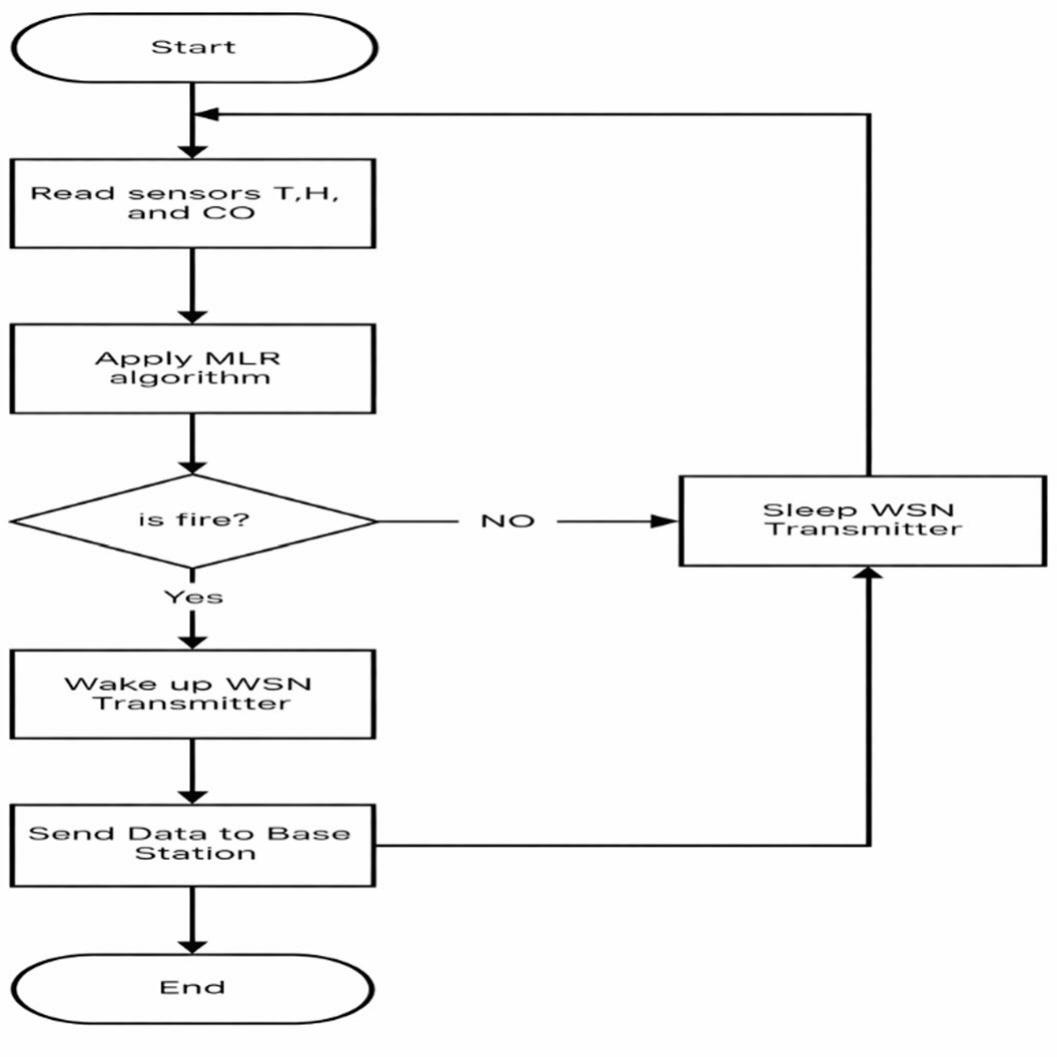
Sensor node flowchart.

**Sensor node:** Temperature, humidity, and carbon monoxide sensors are installed in each of the uniform sensor nodes in addition to the RF transceiver, as shown in Fig. 9. In order to save power and reduce the network traffic, each node operates in sleep mode, but when the likelihood of a fire occurring is high, it communicates immediately to the sink node, where the multiple logistic regression algorithm has been used in the sensor node software for predicting the occurrence of fire based on (MLR) algorithm. The flowchart of the sensor node operation is shown in Fig. 10.

Using labeled sensor data that represented fire and non-fire circumstances, the Multiple Logistic Regression (MLR) model’s coefficients were acquired using an offline training procedure. Temperature (T), relative humidity (H), carbon monoxide (CO), and smoke intensity (S) are the predictor variables. The likelihood of a fire occurring is estimated using the logistic regression model as follows:

18 $$\:F=\frac{1}{{e}^{-(}{b}_{0}+{b}_{1}{x}_{1}+{b}_{2}{x}_{2}+\dots\:+{b}_{n}{x}_{n}}\left\{\begin{array}{c}1\:\to\:fire\:occurrence\:\:\:\:\:\:\:\:\:\:\:\:\:\\\:0\:\to\:fire\:non-occurrence\end{array}\right.$$

The model coefficients (b) are estimated by maximizing the log-likelihood function:

19 $$\:\mathcal{l}\left(b\right)=-\frac{1}{N}\sum\:_{i=1}^{N}[{y}_{i}\mathrm{log}\left({p}_{i}\right)+\left(1-{y}_{i}\right)\mathrm{log}\left(1-{p}_{i}\right)]$$

where y_i_ is the class label observed (1 for fire, 0 for non-fire), and P_i_ is the anticipated likelihood for sample I. Until convergence, the optimization is carried out repeatedly utilizing gradient-based techniques. Following training, the sensor node firmware incorporates the improved coefficients, allowing for lightweight edge calculation of the fire suspicion probability and alarm transmission only upon exceeding the predetermined threshold.

The microcontroller reads the data from the sensor and then works with it. The multiple logistic regression approach is used to test the sensors’ parameters. The nRF24L01 transceiver wakes up and begins sending data to the base station when the fire detection goes over a certain level. If not, the transceiver stays in sleep mode. This action cuts down on traffic on the WSN and increases the lifetime of the node.

**Fig. 11:**
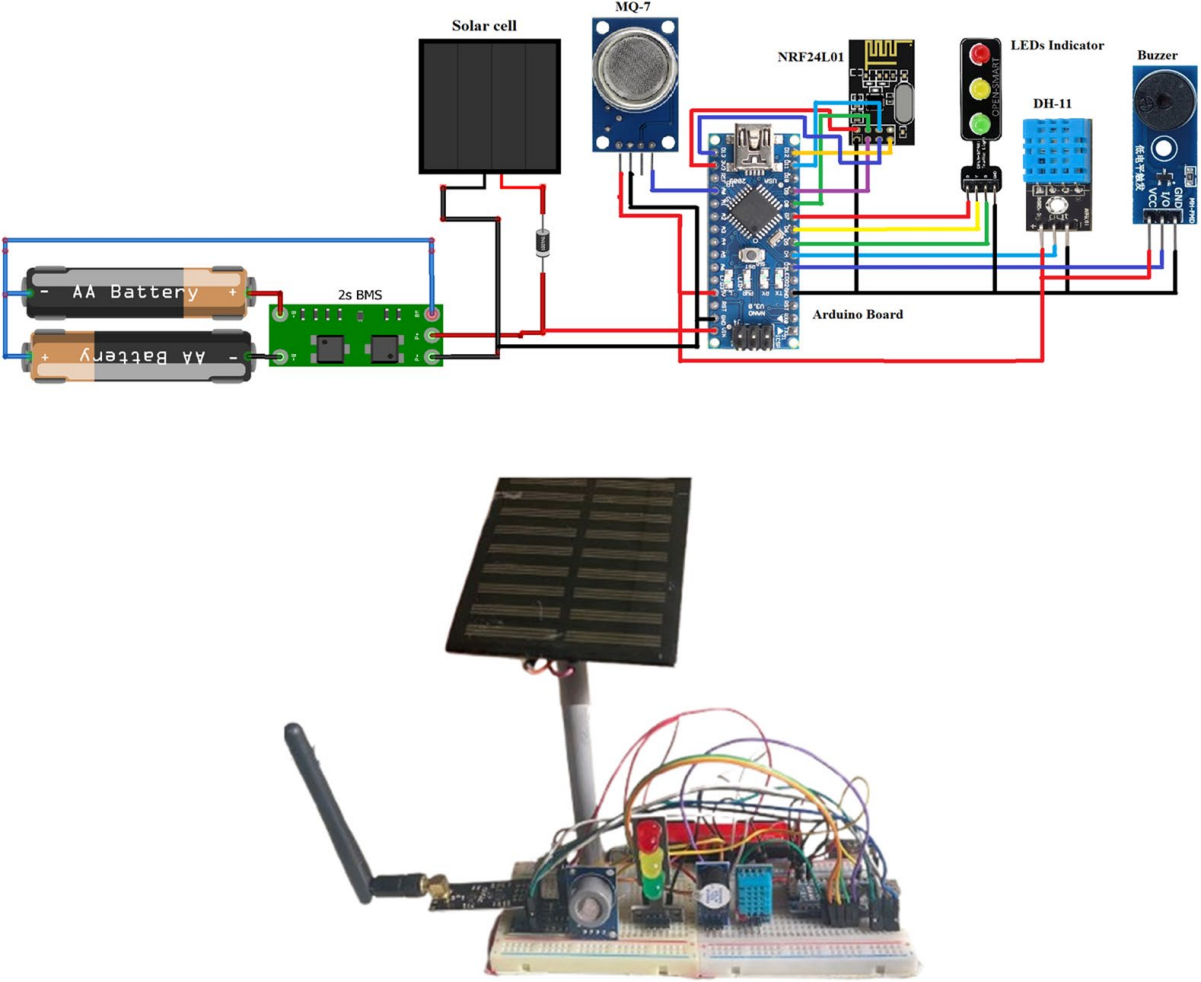
Prototype circuit diagram of the cluster head.

**Fig. 12:**
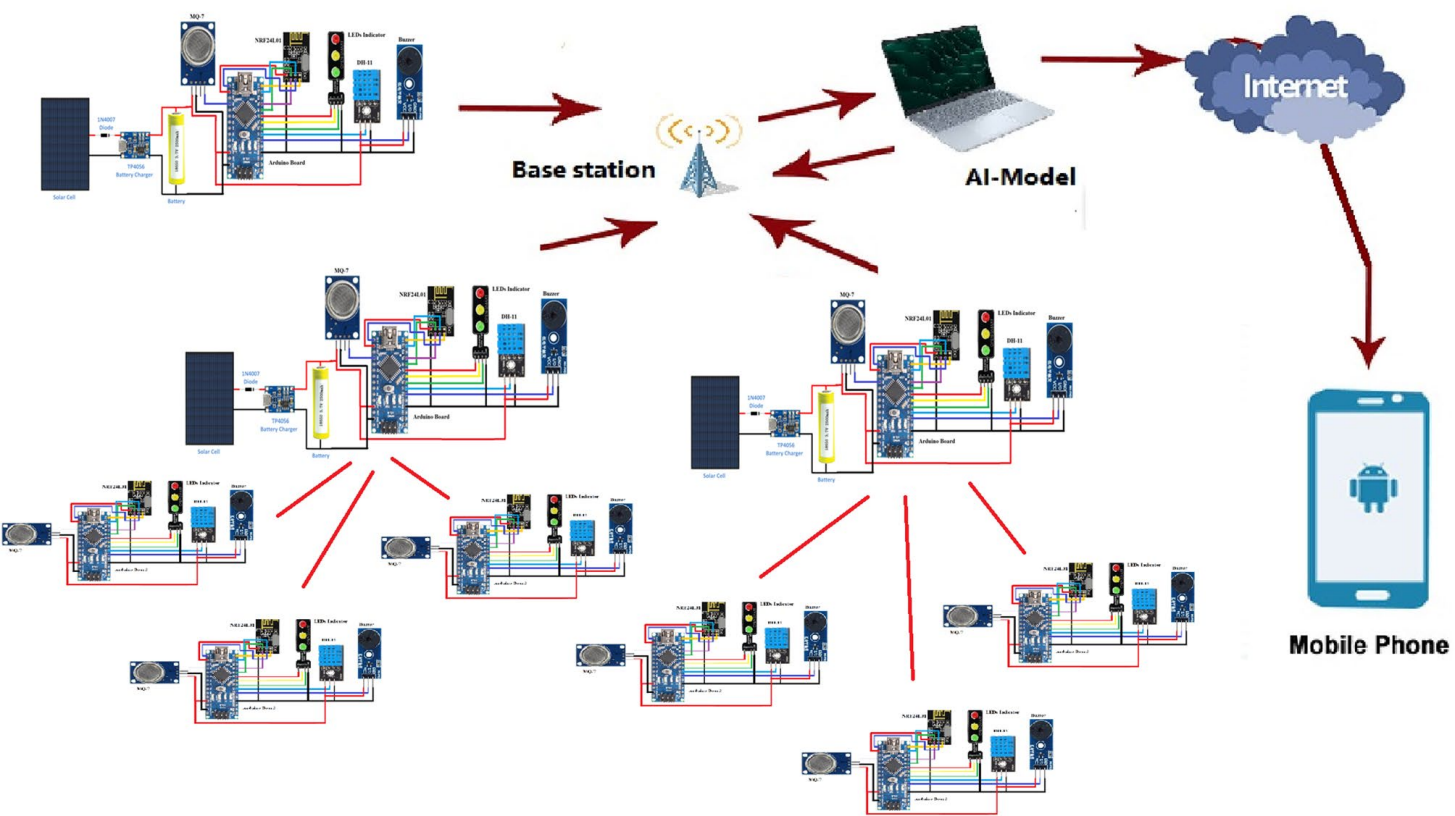
Prototype of the Forest fire detection based on IoT and an AI model.

- **Cluster Head (Sink-node)**: The cluster head functions like other sensor nodes in reading data from sensors, in addition to its other function of connecting to multiple sensor nodes and continuously reading data from the remaining nodes. This requires significant power consumption, and to solve this problem, a solar panel was added to each cluster head, as shown in Fig. 11.

- **Base Station (gateway)**: After the cluster head receives data from the sensor node, it sends the potential fire-related data to the gateway, which contains an RF-XGB AI model. This model then performs a final check of the data to update the cloud database, allowing for notifications to be sent to the public and fire departments via smartphone applications, as shown in Fig. 12.

## Results and discussions

**Fig. 13:**
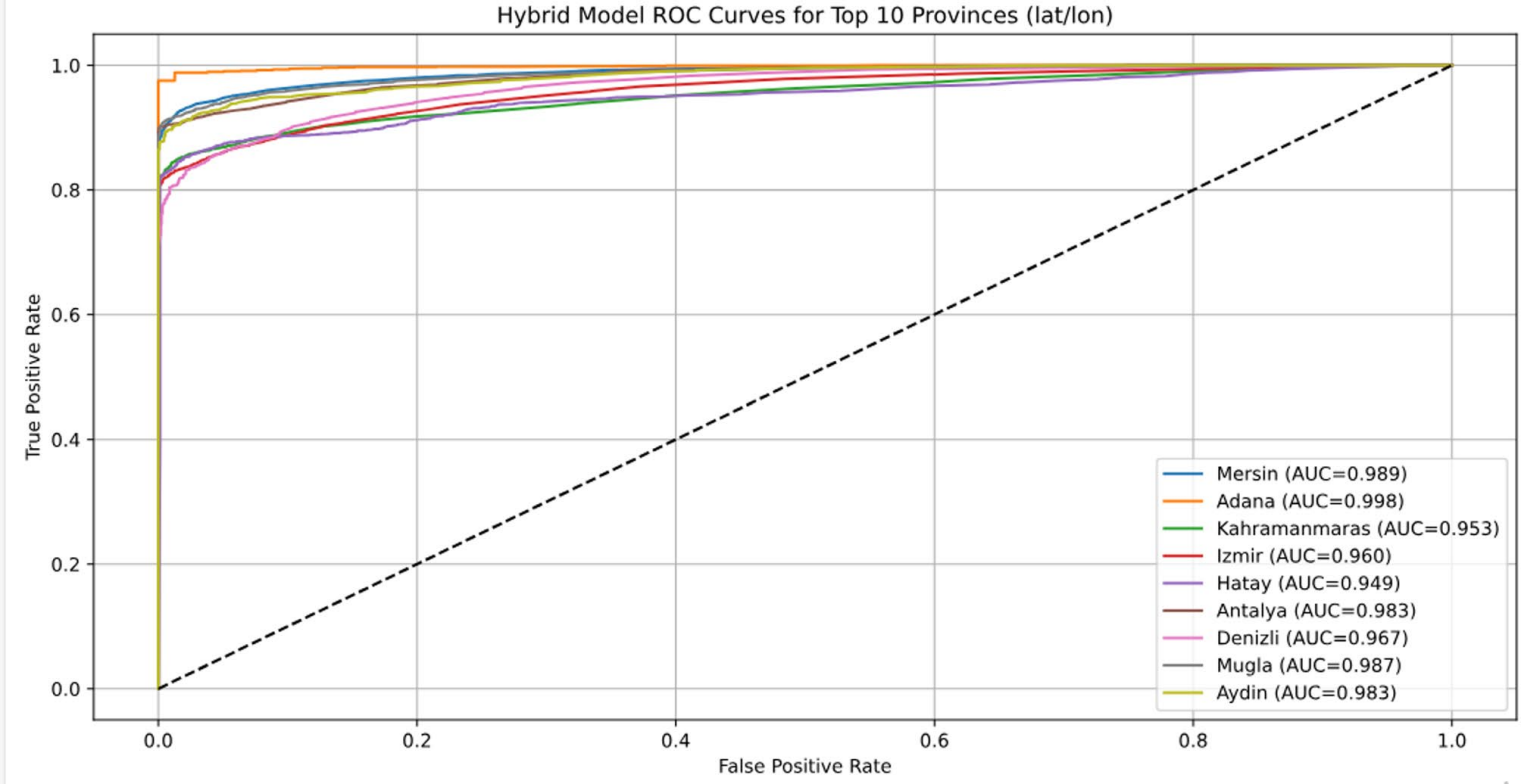
Hybrid RF–XGB ROC curves across top 10 provinces (spatial validation).

Early detection of forest fires is crucial for minimizing environmental and economic losses. With the rapid advancement of artificial intelligence, machine learning techniques can be effectively applied for fire classification using forest fire datasets from the study area in Turkey. In this study, a novel hybrid model (RF-XGB) was proposed, combining the Random Forest and Extreme Gradient Boosting algorithms using a weighted voting prediction strategy. The dataset consists of 450,481 samples collected between 2000 and 2024, including 241,862 fire observations (53.69%) and 208,619 non-fire samples (46.31%). Latitude and longitude coordinates were used to generate 0.5° × 0.5° spatial blocks, and the 10 spatial blocks with the highest fire occurrence were selected for model validation, resulting in 74,645 samples used for modeling. A spatial 10-fold cross-validation strategy was adopted to ensure spatial independence between training and testing data. In each iteration, one spatial block was used as the test set while the remaining blocks were used for training, which helps reduce spatial autocorrelation bias and provides a more realistic evaluation of model generalization across unseen regions. The number of test samples per fold ranged from 4,143 to 14,369. Model performance was evaluated using accuracy, precision, recall, F1-score, Cohen’s kappa coefficient, and ROC–AUC, reported as mean ± standard deviation across folds, as presented in Table 2; Fig. 13.

**Table 2:** Spatial cross-validation results per province.

Fold	Province	Accuracy	Precision	Recall	F1-Score	Kappa	AUC
1	Mersin	0.943	0.984	0.9471	0.9652	0.8076	0.9868
2	Adana	0.9958	0.9981	0.9977	0.9979	0.7095	0.9978
3	Kahramanmaras	0.8878	0.9856	0.8782	0.9288	0.6685	0.9526
4	Izmir	0.8895	0.998	0.8127	0.8959	0.7808	0.9617
5	Hatay	0.8921	0.9314	0.9365	0.9339	0.6405	0.9523
6	Antalya	0.9564	0.9984	0.9024	0.948	0.9106	0.9838
7	Denizli	0.8543	0.9913	0.777	0.8712	0.7092	0.973
8	Mugla	0.9639	0.9939	0.9096	0.9499	0.9218	0.9878
9	Aydin	0.9361	0.9884	0.9044	0.9445	0.8695	0.9816
10	Alanya	0.9854	1	0.92	0.9583	0.9495	0.9861
Mean		0.9304	0.9869	0.8986	0.9394	0.7967	0.9764
±STD		± 0.0472	± 0.0203	± 0.0639	± 0.0355	± 0.1124	± 0.0158
CI		0.8967–0.9641	0.9724–1.0000	0.8529–0.9443	0.9140–0.9648	0.7164–0.8770	0.9651–0.9877

The proposed hybrid model combining Random Forest and XGBoost with confidence-based selection demonstrated strong predictive performance across the ten most fire-prone provinces in Turkey. As shown in Table 1, fold-wise evaluation yielded high accuracy and precision, with mean values of 0.9304 ± 0.0472 and 0.9869 ± 0.0203, respectively, indicating that the model effectively identifies fire occurrences while minimizing false positives. Because of under-detection in certain provinces, such as Denizli and Izmir, which reflected regional differences in fire intensity and sample size, recall was somewhat lower (0.8986 ± 0.0639). Provinces like Antalya, Mugla, and Alanya have exceptionally good F1-scores (0.9394 ± 0.0355) and Cohen’s Kappa (0.7967 ± 0.1124), indicating a balanced performance between accuracy and recall. The model’s ability to reliably differentiate between fire and non-fire events across geographically diverse regions is further supported by its AUC of 0.9764 ± 0.0158. Cross-validation using spatial blocks instead of random splits reduces the impact of spatial autocorrelation and guarantees that the assessment reflects actual generalization performance. Overall, the results suggest that the hybrid approach is robust and suitable for operational wildfire prediction and detection in regions with diverse environmental and meteorological conditions.

**Fig. 14:**
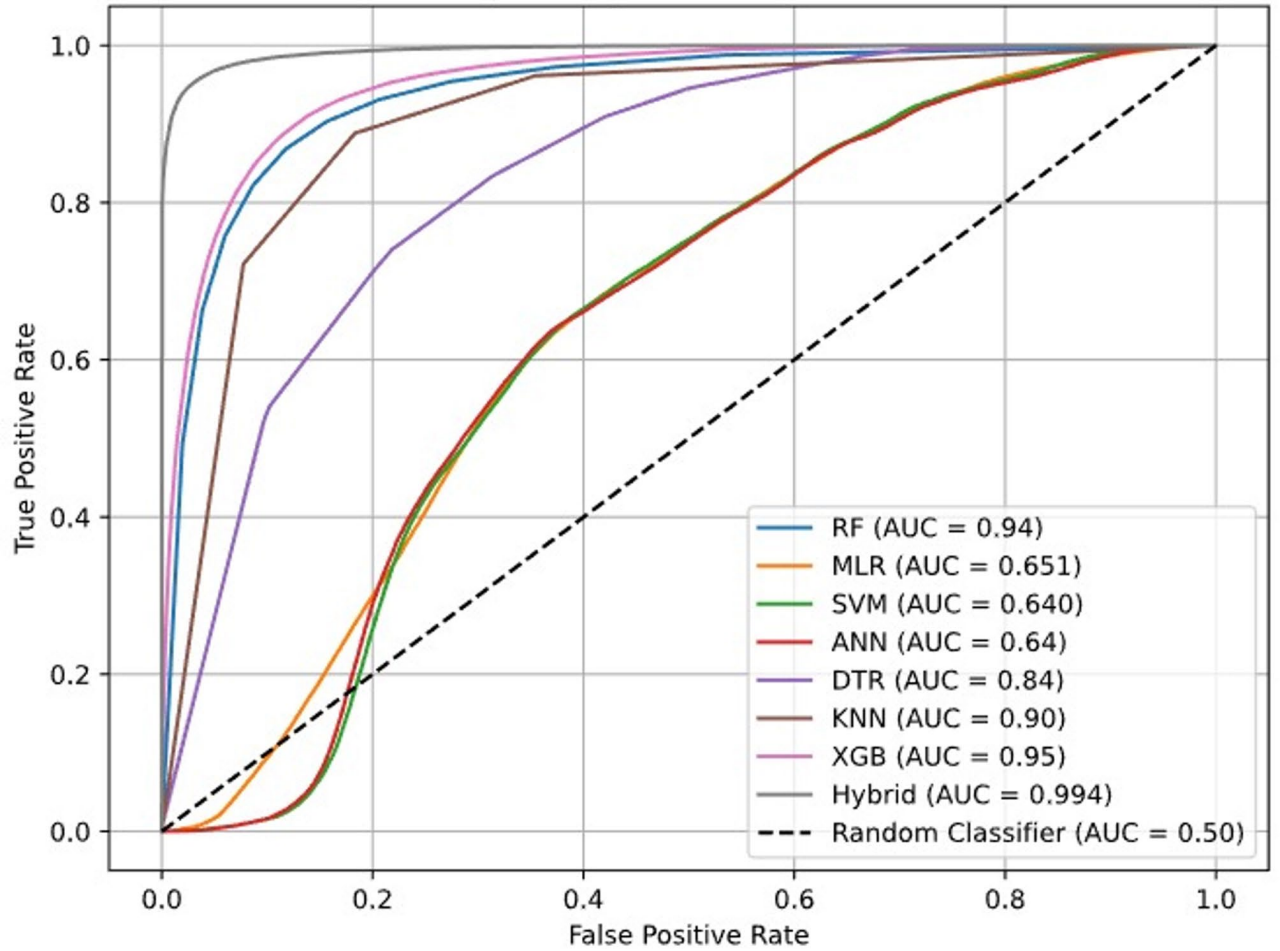
Performance comparison using ROC–AUC for fire prediction models.

The proposed model was compared with several algorithms and demonstrated superior accuracy and a lower error rate, as shown in Table 3, and Fig. 14 of the ROC curves. The hyperparameters of the employed machine learning models were selected based on commonly used empirical settings in similar wildfire prediction studies. Random Forest was configured with 100 trees, while XGBoost was trained using a learning rate of 0.1 and 100 estimators. The neural network classifier utilized a single hidden layer with 100 neurons and 300 maximum iterations. The SVM classifier was implemented using a linear kernel with default regularization, and KNN was configured with five nearest neighbors.

**Table 3:** Performance evaluation of machine learning models for forest fire prediction.

Model	Accuracy	F1 Score	Precision	Recall	AUC	MSE	RMSE	MEAN	STD
RF	0.8753	0.8744	0.8804	0.8685	0.94	0.093636	0.306	0.5108	0.4095
MLR	0.6318	0.6504	0.6187	0.6855	0.651	0.230784	0.4804	0.5015	0.137
ANN	0.6281	0.6494	0.6137	0.6894	0.64	0.232517	0.4822	0.5029	0.1126
KNN	0.8525	0.8575	0.8289	0.8882	0.9	0.116486	0.3413	0.5308	0.4377
DTR	0.76	0.7763	0.7263	0.8338	0.84	0.16024	0.4003	0.5004	0.3002
SVM	0.6313	0.652	0.6169	0.6914	0.64	0.231939	0.4816	0.5014	0.1331
XGB	0.8861	0.8876	0.8749	0.9008	0.95	0.083926	0.2897	0.5015	0.4031
Proposed hybrid model	0.9631	0.9627	0.9734	0.9521	0.994	0.03045	0.1745	0.5163	0.4547

The results of comparing different machine learning models used for forest fire prediction showed the clear superiority of the proposed hybrid model over all individual models. The hybrid model’s high F1 value (0.9627) demonstrated its exceptional ability to identify fire conditions while limiting false alarms, and it also earned the greatest accuracy value (Accuracy = 0.9631) and the best balance between recall and accuracy. The model also recorded the highest ROC–AUC value (0.994), confirming its high performance to distinguish between fire and non-fire conditions. The hybrid model showed a significant degree of convergence between projected and real values, achieving the lowest values for both MSE and RMSE in terms of prediction error. While models like MLR, ANN, and SVM had limited performance, especially in terms of discrimination (AUC), individual models like RF and XGB performed well but still lagged behind the hybrid model.

The AUC measure was used to calculate the Relative Improvement (RI) for each unique model in order to better quantify the performance increase. The hybrid model outperformed the strongest baseline models, Random Forest and XGBoost, by 5.7% and 4.6%, respectively. Significant improvements were seen in comparison to Decision Tree (18.3%) and KNN (10.4%). Interestingly, the hybrid model outperformed MLR, SVM, and ANN by more than 50%, demonstrating its noticeably improved discriminating abilities. These findings support the hybrid framework’s resilience and applicability for wildfire forecasting applications by demonstrating that combining RF and XGB produces consistent and quantifiable performance enhancements across all assessed models.

The suggested hybrid model was compared ten times to the most robust baseline model (XGB). The mean, standard deviation (STD), and 95% CI for important performance indicators are compiled in Table 4. To determine if improvements over the baseline were statistically significant, a paired t-test was used (𝛼 = 0.05).

**Table 4:** Performance of the proposed hybrid model vs. XGB baseline.

Metric	Mean ± STD	95% CI	Baseline	t-value	*p*-value	Significant?
Accuracy	0.9631 ± 0.0472	0.9304–0.996	0.8861	5.16	0.000	Yes
F1-score	0.9627 ± 0.0355	0.9400–0.9854	0.8876	6.69	0.000	Yes
Precision	0.9734 ± 0.0203	0.9580–0.9888	0.8749	15.35	0.000	Yes
Recall	0.9521 ± 0.0639	0.9143–0.9899	0.9008	2.54	0.030	Yes
AUC	0.994 ± 0.0158	0.9822–1.0058	0.9500	8.82	0.000	Yes

The Multiple Logistic Regression (MLR) model is computationally lightweight, making it suitable for application within sensor nodes with limited resources. Although the results of this research showed that MLR performed lower than advanced models in terms of accuracy and discrimination (AUC = 0.651), it offers several important practical advantages in IoT environments. Due to its simple computational architecture, it can be easily integrated into microcontrollers without requiring high processing power. MLR was used in sensor nodes to reduce network traffic and conserve energy.

**Fig. 15:**
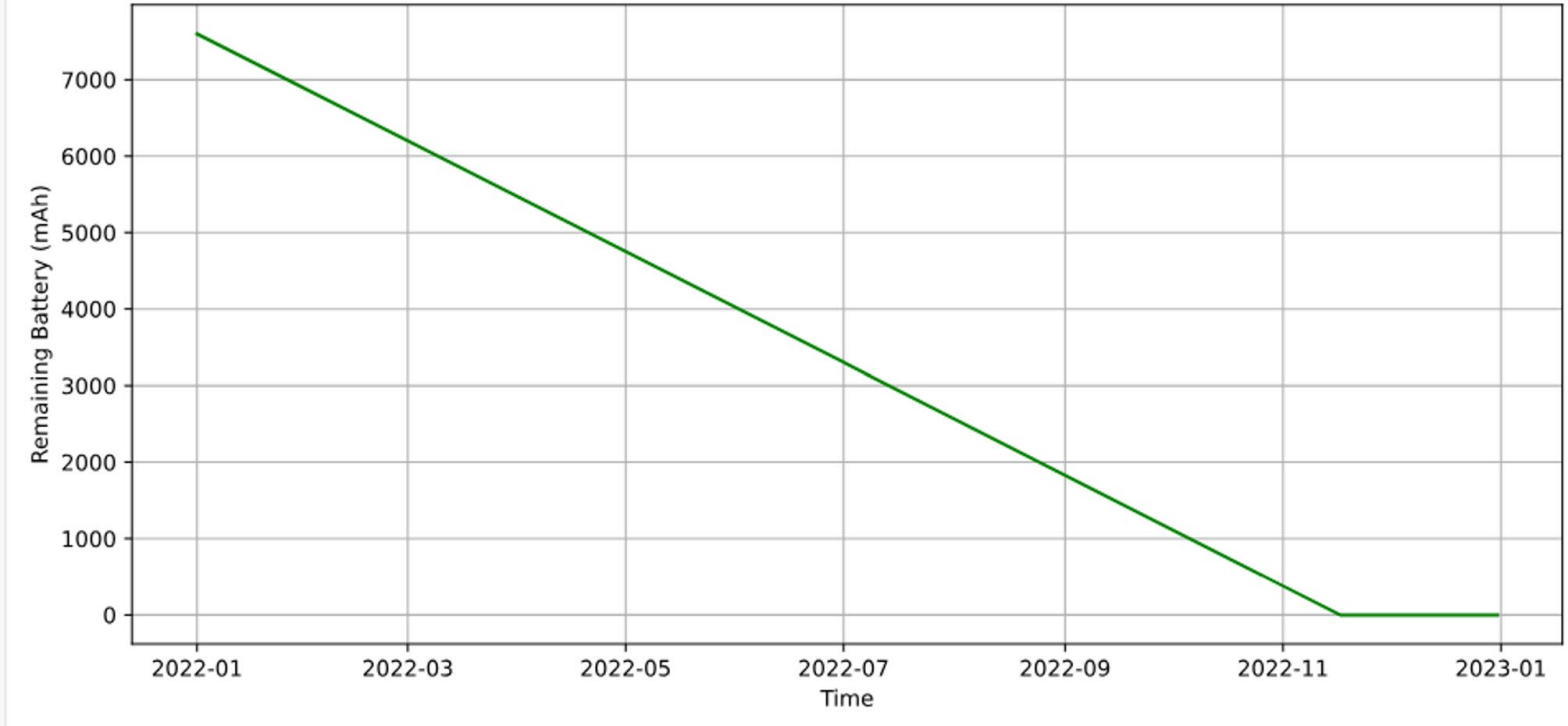
Sensor node lifetime.

**Fig. 16:**
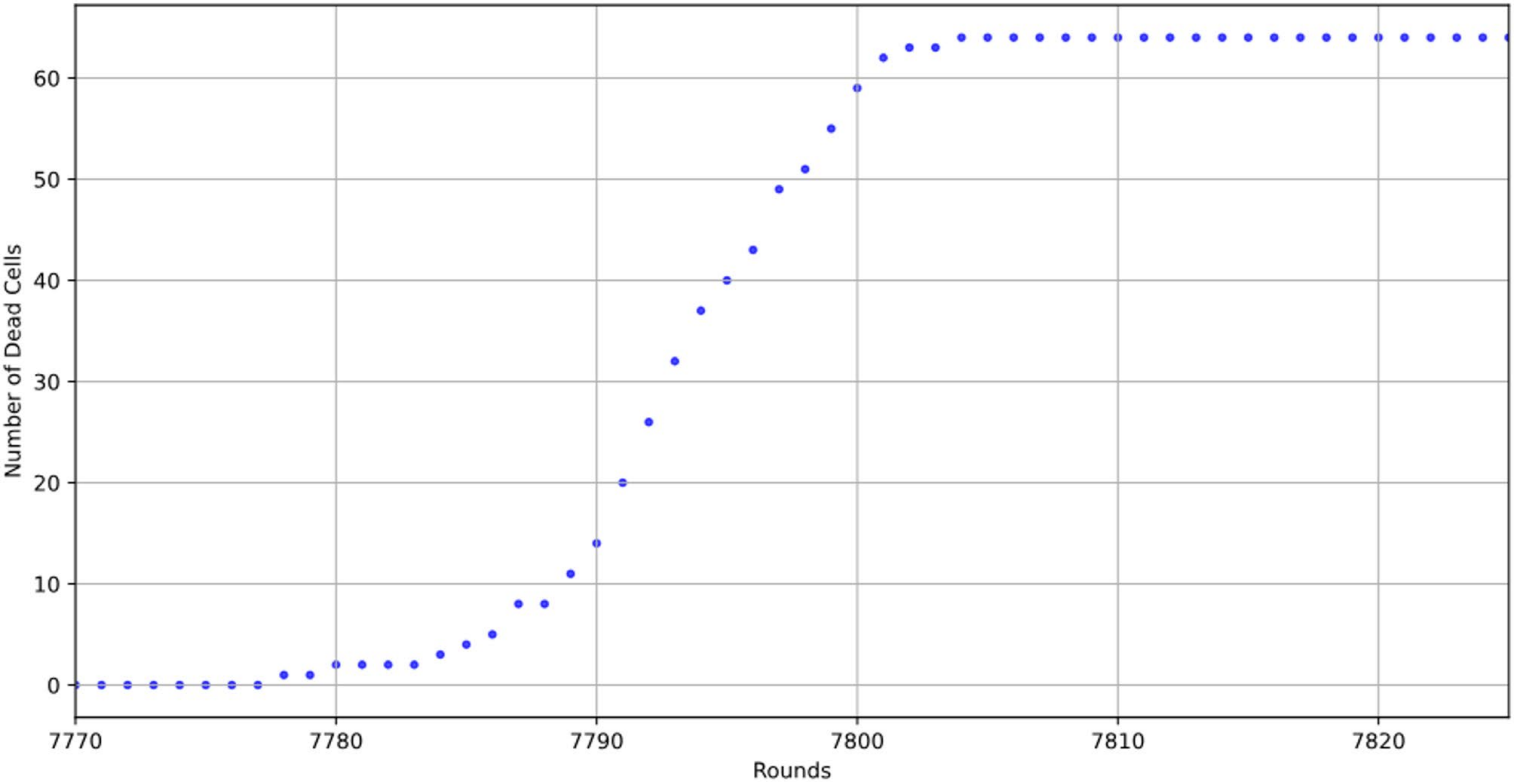
Dead sensor nodes in each round.

The reported node lifetime (up to 11 months) was obtained through analytical energy consumption calculations based on real hardware measurements. Current consumption values were measured for active, idle, and sleep operating modes of the Arduino Nano and communication module, then combined with the battery capacity used in the deployment scenario. Therefore, the reported lifetime reflects a hardware-based estimation rather than a purely theoretical simulation. Figure 15 shows the lifetime of a sensor node, which transmits information only when a fire is detected and otherwise remains in sleep mode. Figure 16 shows the dead nodes in each round.

The Arduino Nano is a popular choice for building sensor nodes due to its ease of programming and readily available components, but it’s not the most energy-efficient. In its active state, the Nano typically draws 25–30 mA at 16 MHz, and with the addition of an MQ-7 sensor (heater), the instantaneous draw rises to approximately 120–180 mA during heating cycles. The nRF24L01 radio module adds about 11–14 mA during transmission and has a very low standby time (~ 0.9 µA) when power-down is used. Therefore, a system built has a short battery life unless power-gating and duty-cycling strategies are implemented, along with strict power policies. The node applies a local multivariable logistic regression (MLR) model to estimate the probability of a fire from sensor readings. To minimize power consumption, the system operates on a cycle that wakes up once an hour to perform measurements and calculations, then immediately returns to sleep mode. If the probability value exceeds a predefined threshold, the node wirelessly transmits data to the sink node via an NRF24L01 module. Under normal circumstances, no transmission occurs, thus minimizing power consumption. This design allows the node to operate for extended periods of up to 11 months using small batteries. The sensor node requires 59 mA when active and 1 mA when in sleep mode, with a total hourly consumption (sleep + wake) of 1.01653 mAh.

**Fig. 17:**
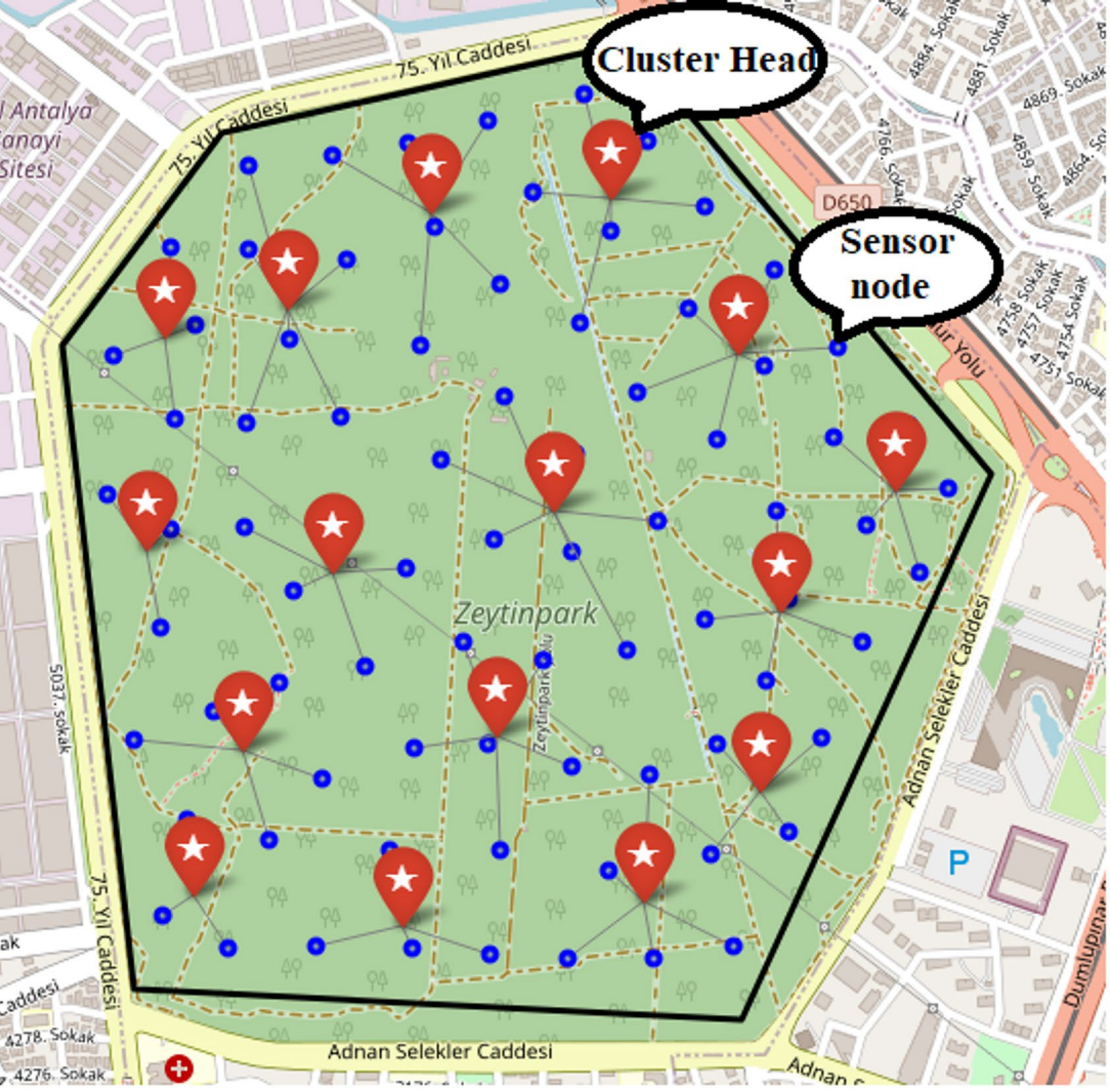
Cluster head and sensor nodes distribution in Zeytinpark.

**Fig. 18:**
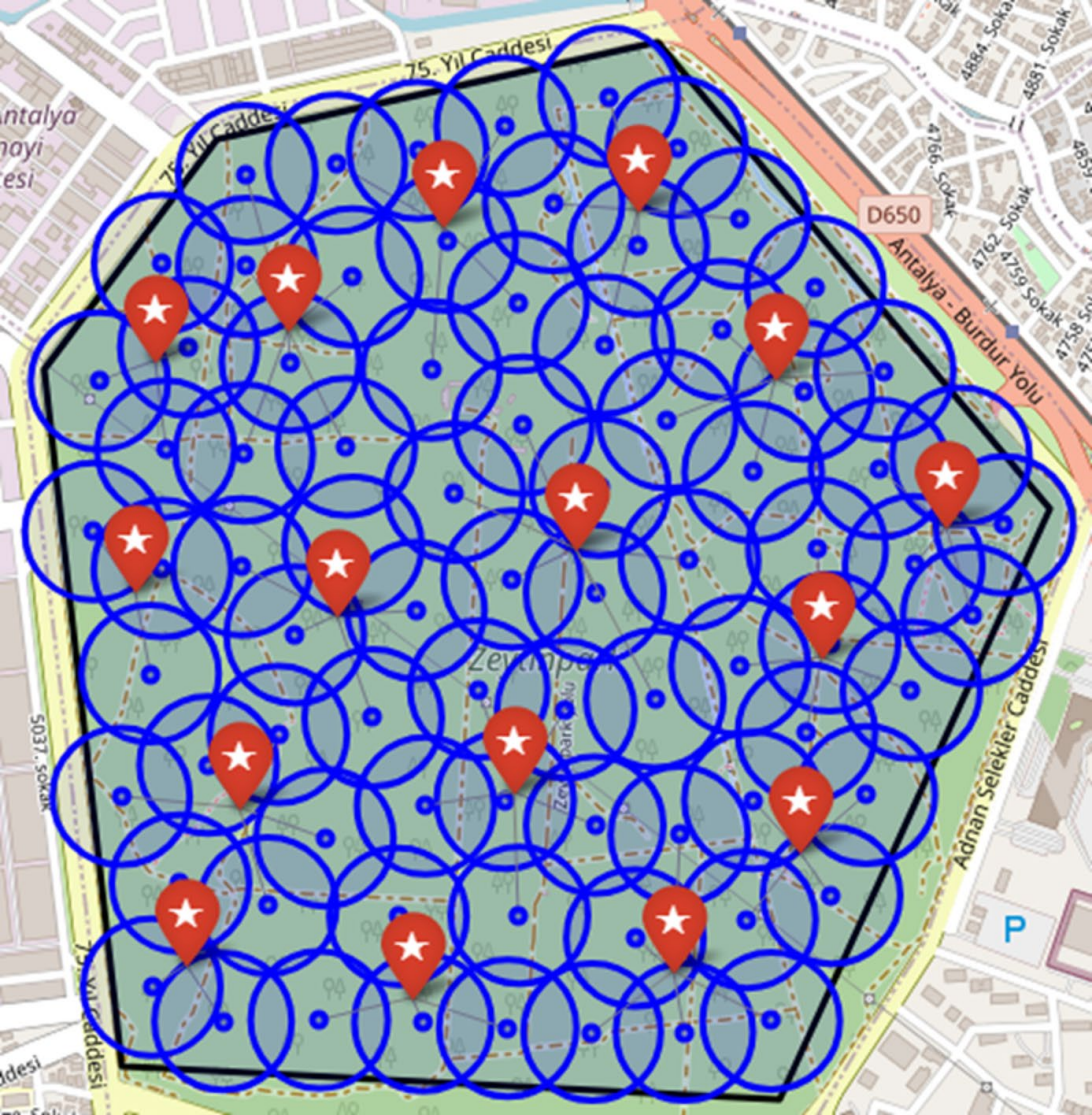
WSN coverage area in Zeytinpark.

Zeytinpark served as a case study for the system’s implementation. The system needed 80 sensor nodes spread using a grid architecture and K-Means algorithm to achieve thorough and balanced park coverage since each sensor node could cover an area of 31,400 m² with a radius of 100 m. The park was separated into equal grid cells, and the node placements were adjusted to be centered inside each block using K-Means within each cell. This reduced interference and made sure that every area of the park was effectively monitored. In order to minimize power consumption and maximize network lifetime, the number of cluster head nodes (CH) for each sub-grid was chosen in order to coordinate data collection and transmission to the control station. As shown in Fig. 17, there were sixteen cluster head nodes. Figure 18 illustrates that the intended area’s network coverage percentage was 95.58%.

**Fig. 19:**
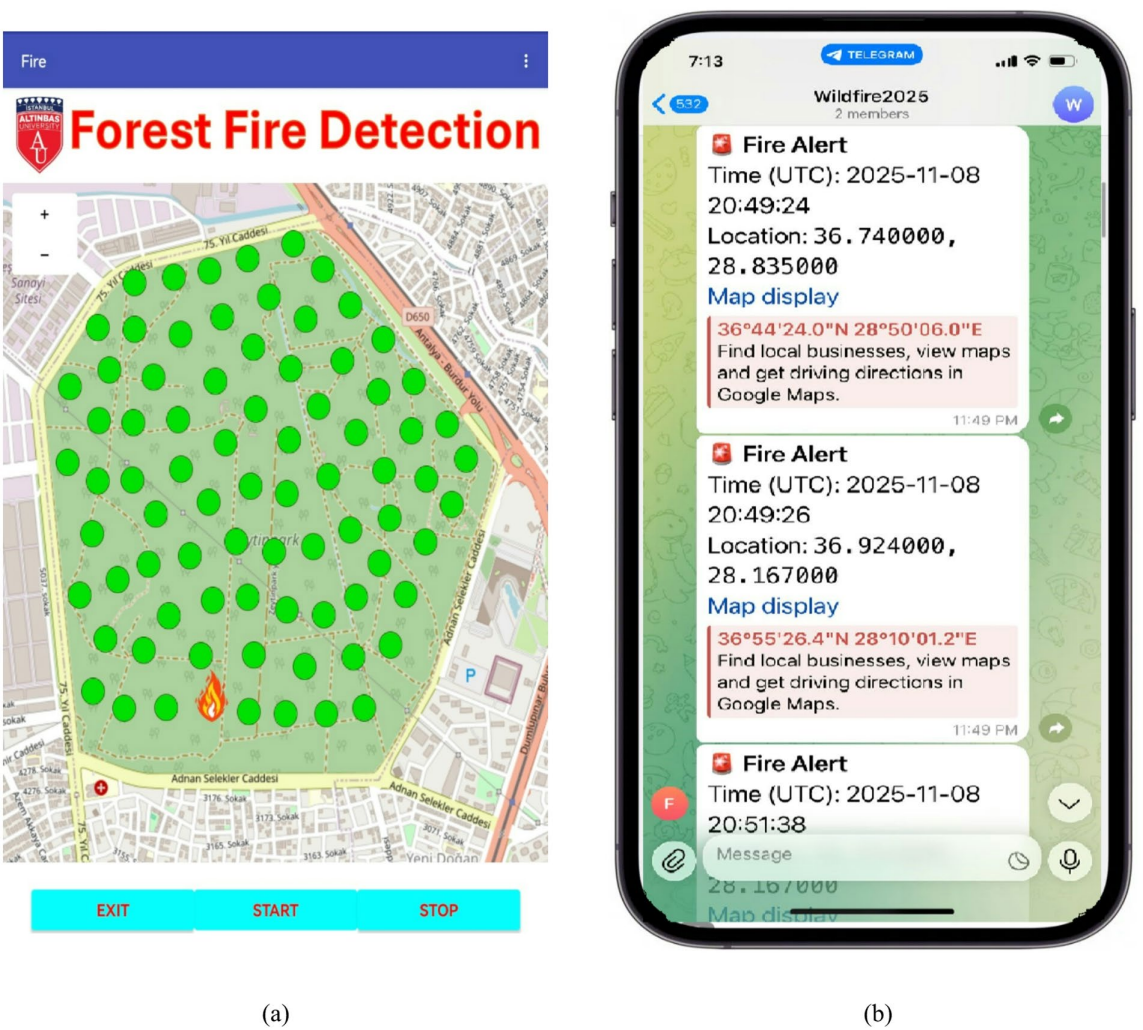
Forest fire cloud notification. (**a**) Fire track application, (**b**) Telegram bot API.

Additionally, the results show how well the integrated system—which combines wireless sensor networks, artificial intelligence, and Internet of Things technologies—handles early forest fire warning situations. The central node (Sink Node), which serves as a bridge between the field network and the sophisticated AI model, receives data instantly when a sensor node identifies a possible fire based on the local MLR model. The hybrid prediction model (RF-XGB) receives this data from the Sink Node and uses it to determine if the scenario is a false alarm or a genuine fire. In the event that a fire is verified, the system sounds a buzzer at each sensor node in the vicinity to set off a local alarm, warning staff and guests to leave the property right away. At the same time, cloud databases are updated, and residents and pertinent authorities are notified instantly by a Tel-egram bot about the location of the fire on an interactive map. Furthermore, as seen in Fig. 19, the information is sent to a specific mobile application for forest fire monitoring, facilitating quick reaction procedures by firefighting crews and pertinent centers.

This integration of IoT, WSN, and artificial intelligence achieves immediate fire detection and rapid incident handling, which contributes to reducing economic losses resulting from the spread of fires, protecting human and animal lives, and preserving biodiversity and vegetation within forests.

When compared to satellite-only monitoring methods, the suggested IoT-based wildfire detection system has benefits. Thermal image processing, which is often used in satellite systems, necessitates computationally demanding analysis and may cause delays because of picture capture and processing time. In addition, satellite monitoring may be expensive and may not always offer real-time fire detection at ground level. On the other hand, near real-time detection from environmental data is made possible by the suggested sensor-based system. However, as wildfire patterns differ depending on meteorological conditions, plant types, and human activity, it is important to apply the trained model to different geographic areas. Therefore, if enough local environmental and fire incidence data are available for retraining, the model may be modified for various locations. The sensing layer may also be affected by limitations of the MQ-7 gas sensor, which requires periodic heating cycles that can introduce fluctuations in measurement stability. In addition, environmental conditions such as temperature and humidity may influence sensor accuracy during long-term deployment.

## Conclusions

This study presented an integrated early forest fire detection and prediction system that combines IoT-based wireless sensor networks with advanced artificial intelligence techniques. A new hybrid model (RF-XGB) was developed and demonstrated superior predictive performance compared to traditional machine learning models. The proposed hybrid Random Forest–XGBoost model, validated through spatial cross-validation across Turkey’s most fire-prone provinces, demonstrates robust and reliable wildfire prediction, making it a promising tool for operational fire risk management and early warning systems. The hybrid model maintained the lowest prediction error (MSE and RMSE) while achieving the best accuracy, F1-score, and ROC–AUC values, confirming its strong performance to discriminate between fire and non-fire scenarios. These findings demonstrate how well Random Forest and Extreme Gradient Boosting work together in a weighted-voting architecture to increase the accuracy of wildfire predictions.

At the sensor-node level, the research also illustrated the usefulness of using the computationally efficient Multiple Logistic Regression (MLR) model. Its simplicity, low processing requirements, and adaptability for microcontroller deployment make it an excellent option for reducing communication burden, lowering network traffic, and saving energy, even if its predictive performance is not as good as that of sophisticated models. With the help of the nRF24L01 module and hourly duty-cycling, the power-optimized design greatly increased node lifespan, enabling continuous operation for up to 11 months on tiny batteries.

The scalability and effectiveness of the system were validated by the field installation in Zeytinpark. With 80 sensor nodes and 16 cluster heads, the park’s coverage was balanced thanks to the combination of grid-based deployment and K-Means clustering, which produced a 95.58% coverage rate. Near real-time fire detection and decision-making were made possible by the hybrid AI model’s integration with IoT infrastructure. Sensor data is sent to the sink node upon detection of a possible fire, where the RF-XGB model processes it to verify the warning. Multiple levels of notifications are triggered upon approval, including interactive fire-location mapping for firefighting personnel, cloud database updates, Telegram alerts, mobile app notifications, and local alarms via buzzers installed on nodes.

Overall, the findings show that a strong foundation for real-time wildfire monitoring is provided by the combination of IoT, wireless sensor networks, and AI-based prediction models. Faster reaction, less financial harm, and the preservation of human life, animal, and forest biodiversity are all made possible by the system’s quick detection, precise categorization, and effective alarm dissemination. The suggested method is a potential first step in creating energy-efficient, scalable, and intelligent forest fire detection systems that may be used in the real world. Next-generation intelligent wildfire monitoring systems built on adaptive autonomous learning architectures will be the main focus of future research. Combining self-evolving hybrid ensemble models that can continually update prediction limits without needing total retraining with real-time streaming environmental data is one exciting avenue. Furthermore, explainable artificial intelligence (XAI) procedures will be investigated in order to enhance operational emergency management and increase transparency in fire risk decision-making.

## Data Availability

https://firms.modaps.eosdis.nasa.gov/map/#d:24hrs;@17.5,-0.3,2.4z; https://forest-fire.emergency.copernicus.eu/.
